# Hyphal Development in *Candida albicans* Requires Two Temporally Linked Changes in Promoter Chromatin for Initiation and Maintenance

**DOI:** 10.1371/journal.pbio.1001105

**Published:** 2011-07-19

**Authors:** Yang Lu, Chang Su, Allen Wang, Haoping Liu

**Affiliations:** Department of Biological Chemistry, University of California, Irvine, California, United States of America; Duke University Medical Center, United States of America

## Abstract

Phenotypic plasticity is common in development. For *Candida albicans*, the most common cause of invasive fungal infections in humans, morphological plasticity is its defining feature and is critical for its pathogenesis. Unlike other fungal pathogens that exist primarily in either yeast or hyphal forms, *C. albicans* is able to switch reversibly between yeast and hyphal growth forms in response to environmental cues. Although many regulators have been found involved in hyphal development, the mechanisms of regulating hyphal development and plasticity of dimorphism remain unclear. Here we show that hyphal development involves two sequential regulations of the promoter chromatin of hypha-specific genes. Initiation requires a rapid but temporary disappearance of the Nrg1 transcriptional repressor of hyphal morphogenesis via activation of the cAMP-PKA pathway. Maintenance requires promoter recruitment of Hda1 histone deacetylase under reduced Tor1 (target of rapamycin) signaling. Hda1 deacetylates a subunit of the NuA4 histone acetyltransferase module, leading to eviction of the NuA4 acetyltransferase module and blockage of Nrg1 access to promoters of hypha-specific genes. Promoter recruitment of Hda1 for hyphal maintenance happens only during the period when Nrg1 is gone. The sequential regulation of hyphal development by the activation of the cAMP-PKA pathway and reduced Tor1 signaling provides a molecular mechanism for plasticity of dimorphism and how *C. albicans* adapts to the varied host environments in pathogenesis. Such temporally linked regulation of promoter chromatin by different signaling pathways provides a unique mechanism for integrating multiple signals during development and cell fate specification.

## Author Summary

Many organisms are able to change their phenotype in response to changes in the environment, a phenomenon referred to as plasticity. *Candida albicans*, a major opportunistic fungal pathogen of humans, can undergo reversible morphological changes between yeast (spherical) and hyphal (filamentous) forms of growth in response to environmental cues. This morphological plasticity is essential for its pathogenesis and survival in its hosts. In this study, we show that hyphal development is initiated and maintained by two major nutrient-sensing cellular growth pathways that act by removing the inhibition provided by the transcriptional repressor Nrg1. While initiation requires a rapid but temporary disappearance of Nrg1 via activation of the cAMP-dependent protein kinase A pathway, maintenance requires the recruitment to promoters of the Hda1 histone deacetylase under conditions of reduced signaling by the target of rapamycin (TOR) kinase, leading to chromatin remodeling that blocks Nrg1 access to the promoters of hypha-specific genes. We observed that recruitment of Hda1 to promoters happens only during the time window when Nrg1 is absent. Such temporally linked regulation of promoter chromatin by different signaling pathways provides a unique mechanism for integrating multiple signals in the regulation of gene expression and phenotypic plasticity during development and cell fate specification.

## Introduction

Many organisms or cells are able to alter their phenotype or developmental fate in response to changes in their environment, a phenomenon referred to as plasticity. Plasticity is seen in a broad range of biological systems from embryo development to memory formation where long-lasting effects on gene expression outlive an initial transient signal. The dynamic process of cell fate specification is determined by a network of regulatory genes. The architecture of the network defines the temporal order of specification events. Therefore, understanding temporal dynamic regulation of gene expression in response to extracellular signals is critical for our comprehension of cell fate specification and plasticity.

Morphological plasticity is a defining feature of *Candida albicans*, a major opportunistic fungal pathogen of humans [1]. *C. albicans* resides as harmless commensal flora in the gastrointestinal tract and mucosal membranes of healthy individuals, but when the host immune system is suppressed, the fungus can disseminate and cause systemic infections. Unlike many other pathogenic fungi that exist primarily in either yeast or hyphal forms and infect a specific organ, *C. albicans* is able to undergo reversible morphological changes between yeast, pseudohyphal, and hyphal forms of growth in response to environmental cues and can successfully infect many different anatomical sites of the human host. Its morphological plasticity is the most important virulence attribute of *C. albicans* [2]. Hyphae have invasive properties that can promote tissue penetration and escape from immune cells, whereas yeast cells are suited for dissemination in the bloodstream. Several of the genes that are specifically expressed in hyphae encode virulence factors. For example, *HWP1*, *ALS3*, and *RBT5* encode cell wall proteins that are important for adhesion to host cells and iron acquisition from the host [3]–[6].

Several signal transduction pathways are involved in the regulation of hyphal development. Among them, the cAMP-dependent protein kinase A (PKA) pathway plays an essential role in hyphal morphogenesis and virulence [7]. The adenylate cyclase Cyr1 and its associated protein are indispensable for hyphal growth under all conditions [8]–[10]. Tpk1 and Tpk2 are catalytic subunits of PKA; each plays distinct functions in hyphal development [11]–[13]. Efg1 and Flo8, two transcription regulators essential for hyphal development and virulence [14]–[16], are implicated to function downstream of the cAMP-PKA pathway [17],[18]. The hyphal transcriptional program is repressed by Tup1 through sequence-specific DNA-binding proteins [19]–[27], of which Nrg1 plays a major role. *nrg1* mutant cells are constitutively filamentous under all conditions, similar to *tup1* cells [25],[26]. Ectopic expression of *NRG1* inhibits hyphal filamentation in all growth conditions [28],[29]. Although molecular genetic analyses have identified a number of key transcriptional regulators of hyphal morphogenesis, our understanding of the transcriptional regulation that governs the yeast-to-hypha transition remains rudimentary.

The yeast-to-hypha transition can be induced by a wide range of media and environmental conditions in vitro [1]. Serum in combination with a rise in temperature to 37°C gives the most robust induction of hyphae. Simple inoculation of stationary cells into fresh medium at 37°C is also a powerful but transient inducer of hyphae [30]. Several induction signals are transmitted through Cyr1, including CO_2_/HCO_3_ *^−^* and peptidoglycan found in serum, as well as a rise in temperature [31]–[33]. Cyr1 is also regulated by Ras1, Ras2, and Gpa2 in response to nutrients [34]–[38]. Farnesol, a quorum-sensing molecule secreted to the medium by *C. albicans* cells as a cell density signal [39], exerts its inhibitory effects on germ-tube formation through Ras1-Cyr1 [40]. However, many widely used hyphal-inducing media are poor in nitrogen and carbon sources that are not favorable for the activation of the cAMP-PKA pathway, raising the question of how *C. albicans* can undergo hyphal development in both rich and poor media. The target of rapamycin (TOR) protein kinase pathway is another major nutrient-sensing pathway conserved in *C. albicans* [41]. Rapamycin can both inhibit hyphal formation on solid medium and promote cell aggregation in liquid medium in *C. albicans* [41],[42], but it does not induce yeast-to-hyphal development.

Transcriptional activation or repression in response to external stimuli is often mediated through dynamic changes in promoter chromatin structure. This regulation occurs at many different levels, including posttranslational modification of histones [43], chromatin remodeling [44], incorporation of histone variants [45], and cotranscriptional chromatin disassembly and assembly [46],[47]. All of these dynamic processes work in concert to establish or alter the local properties of chromatin, although the relative importance and order of these processes vary at each individual promoter. Histone acetyltransferases (HATs) and deacetylases (HDACs) play important roles in regulating chromatin structure and transcription [48]. Histone acetylation is a dynamic reversible process, and the balance of histone acetylation is important for proper cellular functions [49]. We have previously shown a dynamic nucleosomal H4 acetylation at the promoters of hypha-specific genes during hyphal induction [50]. H4 histone acetyltransferase complex NuA4 is recruited to promoters in both yeast and hyphal forms and is required for the induction of hyphal genes [50]. Here we show that the dynamic increase and decrease in H4 acetylation at the promoters during hyphal induction correlates with the sequential dissociation and association of two different HDACs, Rpd3 and Hda1, respectively. Concomitant with the dissociation of Rpd3 upon hyphal initiation, Nrg1 protein disappears from cells in response to the activation of the cAMP-PKA pathway, a step essential for hyphal initiation. Although Nrg1 protein returns after hyphal initiation, promoter recruitment of Hda1 under reduced Tor1 signaling leads to deacetylation and eviction of the NuA4 HAT module, nucleosome reassembly, and inhibition of Nrg1 access to the promoters of hypha-specific genes, a step essential for sustained hyphal development. Importantly, disappearance of Nrg1 during hyphal initiation is a prerequisite for promoter recruitment of Hda1. The sequential regulation of hyphal development by the activation of the cAMP-PKA pathway and reduced Tor1 signaling provides a molecular mechanism for the plasticity of dimorphism and how *C. albicans* adapts to the varied host environments in pathogenesis.

## Results

### Yeast-to-Hyphal Development Involves Two Temporally Related Phases of Removing Nrg1 Inhibition

**Figure 1:**
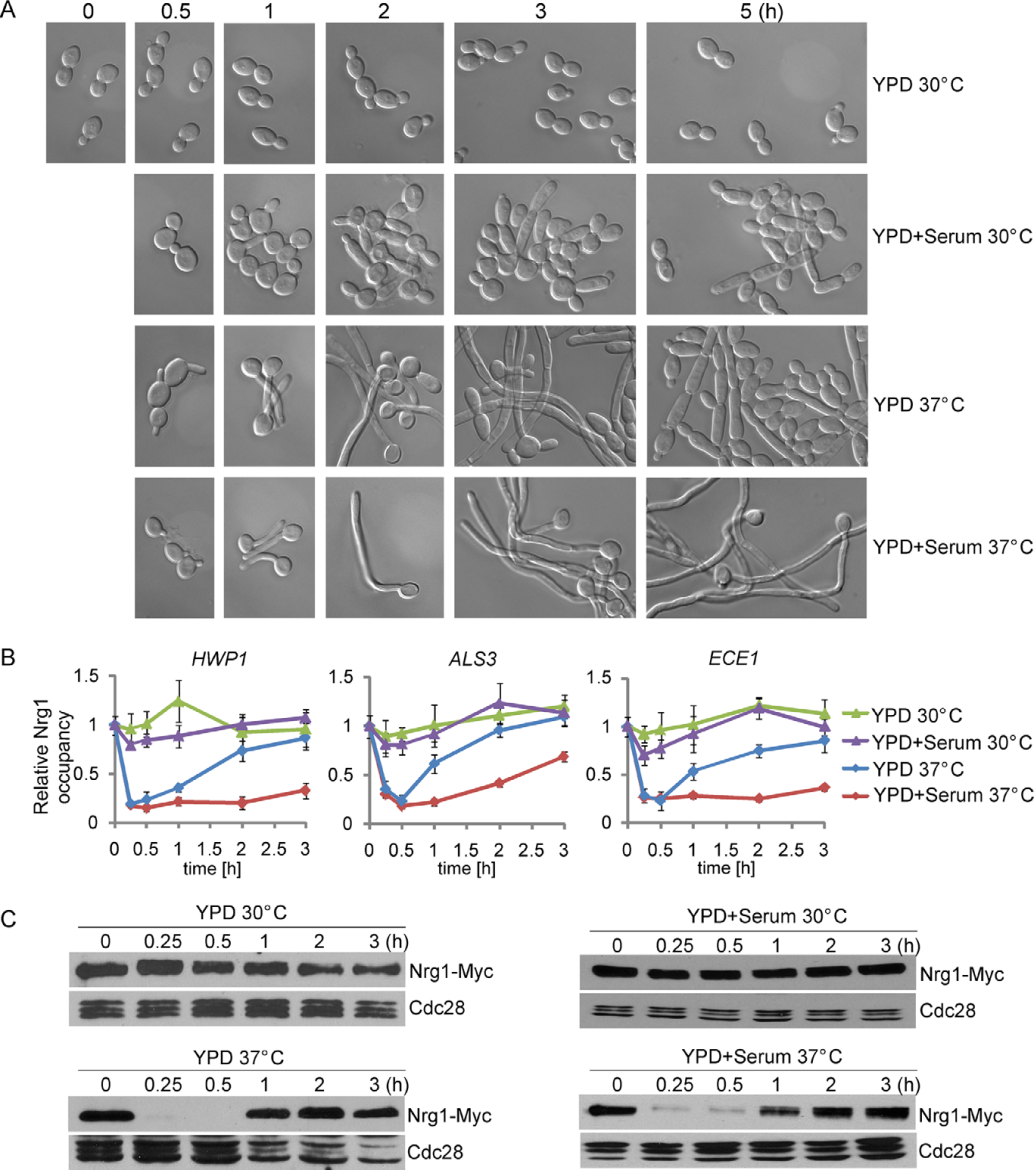
Initiation and maintenance of hyphal development by removing Nrg1 inhibition. An overnight culture of wild-type cells carrying Nrg1-Myc (HLY3922) were diluted 1∶50 into the indicated medium at 30°C or 37°C in the presence or absence of 10% serum, and cells were collected at 0 min, 15 min, 30 min, 1 h, 2 h, 3 h, and 5 h for cell morphology (A), ChIP (B), and Western blot (C) experiments. ChIP DNA were quantitated by qPCR with primers at the UAS regions of *HWP1*, *ALS3*, and *ECE1*, as described [50]. The *ADE2* coding region was used as a control. The Nrg1-Myc enrichment is presented as a ratio of *HWP1*, *ALS3*, or *ECE1* IP (bound/input) versus the control IP (bound/input). The 0 h values on *HWP1* were set to 1.00. The ChIP data show the average of three independent qPCR data with error bars representing the SEM. Western analysis was carried out using a peroxidase-conjugated anti-c-myc (Roche Diagnostics) antibody to assess levels of Nrg1-myc, and with an anti-PSTAIRE (Cdc28, Santa Cruz) antibody for loading control.

The transcription factor Nrg1 plays an essential role in repressing hyphal development [22],[25],[26],[51]. The significance of Nrg1 is underscored by recent phenotypic profiling of 143 transcriptional regulator knockout mutants, where only *nrg1* and *tup1* mutants are filamentous under all conditions examined [52]. Relief of the transcriptional repression by Nrg1-Tup1 may be the essential and regulated step for the activation of the hyphal transcriptional program under all conditions. Indeed, *NRG1* transcript level has been shown to be reduced in hyphae [22],[25],[26],[53]. However, how Nrg1 is regulated at the protein level and whether Nrg1 directly binds to the promoters of hypha-specific genes is not clear. By chromatin immunoprecipitation (ChIP) of C-terminal Myc-tagged Nrg1, we found that Nrg1 was at the promoters of hypha-specific genes *HWP1*, *ALS3*, and *ECE1* during yeast growth (Figure 1). We next analyzed the levels of promoter-bound Nrg1 during yeast to hyphal morphological development under four related conditions in YPD medium that induce three distinctive developmental responses (Figure 1A). Cells inoculated to YPD with or without serum at 30°C remain in the yeast growth form. Cells inoculated into YPD at 37°C in the absence of serum show normal germ tube formation, but are unable to maintain hyphal development. Serum, in combination with a shift in temperature to 37°C, induces robust and sustained hyphal growth. By a time course ChIP, we found that, upon hyphal induction, Nrg1 dissociated rapidly from the promoters and remained at low levels during hyphal elongation in YPD with serum (Figure 1B). In contrast, levels of promoter-associated Nrg1 recovered after the initial decrease under the condition of YPD without serum, which correlated with the conversion to the yeast growth phase around 3 h after the induction (Figure 1A and 1B). Unexpectedly, Western analyses indicated that Nrg1 protein levels decreased sharply at 15 and 30 min upon hyphal induction at 37°C coinciding with germ tube formation. However, the Nrg1 level recovered after 1 h of hyphal induction (Figure 1C). Serum was not required for the disappearance of Nrg1, but was critical for sustained hyphal development and excluding Nrg1 from promoters during hyphal elongation (Figure 1). The Western analysis of cells after 3 h of hyphal induction could not discriminate Nrg1 protein from apical cells and subapical cells. It is possible that Nrg1 protein was absent in the apical cell of each hypha and, therefore, allowed sustained hyphal growth from the apical cells. We determined Nrg1 localization by immunofluorescence in hyphal cells after 5 h of growth in YPD with serum at 37°C and found Nrg1 in the nucleus of both apical and subapical cells (Figure S1), excluding this possibility. Our data suggest that hyphal development involves two phases of releasing Nrg1 inhibition: the first for initiation and the second for maintenance. We suggest that initiation requires a transient down-regulation of Nrg1 protein level, while maintenance requires a regulation that prevents Nrg1 from binding at the promoters of hypha-specific genes.

### The cAMP-PKA Pathway Is Required for the Down-Regulation of Nrg1 for Hyphal Initiation

**Figure 2:**
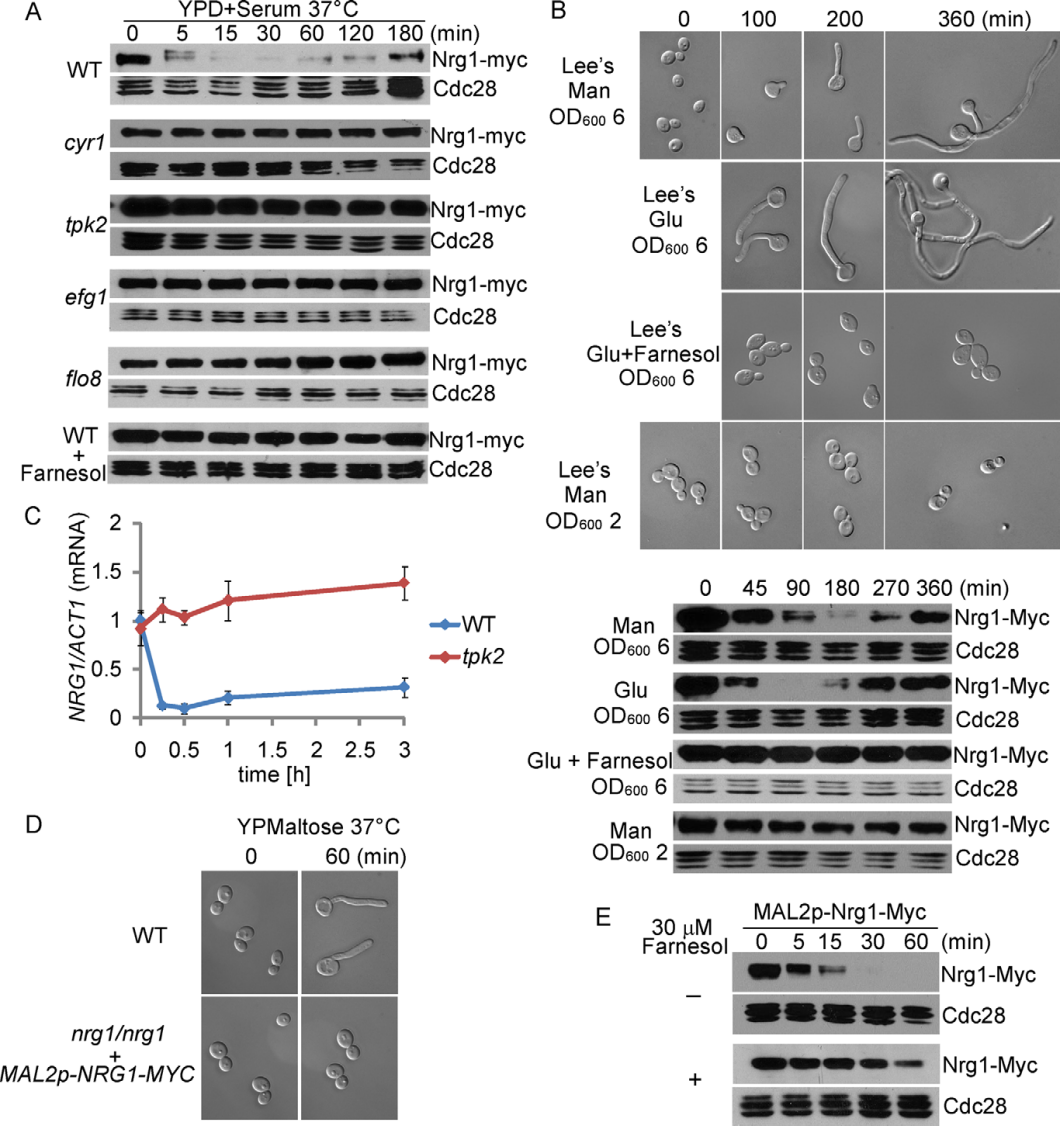
Hyphal initiation requires cAMP-PKA dependent down-regulation of Nrg1 protein. (A) Components of the cAMP-PKA pathway and release from farnesol inhibition are required for down-regulation of Nrg1 protein during hyphal initiation. Western analysis of Nrg1-Myc during hyphal induction in wild-type, *cyr1*, *tpk2*, *efg1*, and *flo8* mutants. Cells of wild type and mutants carrying Nrg1-Myc were diluted 1∶100 into fresh YPD+10% serum medium at 37°C with or without 30 µM farnesol as indicated. (B) Cell morphology and Western blot analysis of wild-type cells carrying Nrg1-myc. Cells from overnight cultures of the indicated OD_600_ were diluted 1∶100 into Lee's media with either mannitol or glucose. Adding 30 µM farnesol into Lee's glucose medium blocked Nrg1 down-regulation and hyphal development. (C) Tpk2 is essential for transcriptional down-regulation of *NRG1* during hyphal induction. *NRG1* mRNA levels were determined by qRT-PCR in WT and *tpk2/tpk2* strains over a time course of hyphal induction (YPD+10% Serum). The signal obtained from *ACT1* mRNA was used as a loading control for normalization. The data show the average of three independent qRT-PCR experiments with error bars representing the SEM. (D) Constitutive expression of Nrg1 blocks germ tube formation. Cells of wild-type and *nrg1/nrg1* carrying Nrg1-Myc under the *MAL2* promoter were grown in YPMaltose medium at 30°C overnight and were diluted at 1∶20 into pre-warmed YPMaltose medium at 37°C. (E) Nrg1 protein stability by promoter-shutdown. Western or wild-type cells carrying Nrg1-Myc under the *MAL2* promoter inoculated from overnight culture into fresh YPD medium at 30°C with and without 30 µM farnesol.

Several lines of evidence suggest that the rapid and temporary disappearance of Nrg1 protein upon hyphal induction is regulated by the cAMP-PKA pathway. First, we found that Nrg1 disappearance required the cAMP-PKA pathway as Nrg1 protein level did not show an obvious reduction in *cyr1* and *tpk2* mutants (Figure 2A). Similarly, no obvious change in Nrg1 level was observed in an *efg1* or *flo8* mutant (Figure 2A). Second, the shift to 37°C during hyphal induction was critical for the observed Nrg1 disappearance (Figure 1C). This is consistent with the report that Hsp90 regulates hyphal development via Cyr1 in response to a shift in temperature [33]. Third, the major quorum-sensing molecule farnesol, at a physiological concentration, completely blocked the down-regulation of Nrg1 (Figure 2A). Farnesol is reported to exert its inhibitory effects on hyphal initiation through Cyr1 [39],[40]. Consistent with the inhibitory effect of farnesol on the down-regulation of Nrg1, different folds of dilution during inoculation could lead to different durations of Nrg1 down-regulation (Figure 1C versus Figure 2A). We also observed that hyphal initiation correlated with Nrg1 disappearance in all liquid media, including nutrient-poor media (Figure 2B). However, hyphal initiation in a poor medium required inoculation of cells from a saturated overnight culture. This result is consistent with the report that release from quorum-sensing molecules, such as farnesol, triggers the yeast-to-hypha transition at 37°C independent of the inoculation media [30]. The timing of hyphal initiation was slower in medium with mannitol than that with glucose (Figure 2B), consistent with the activation of the cAMP pathway by glucose in addition to the release of inhibition from farnesol. Together, our observations suggest that hyphal initiation involves a down-regulation of Nrg1 protein in response to the activation of the cAMP-PKA pathway by a combination of temperature shift, release from inhibition by farnesol, and nutrients in rich media.

The *NRG1* transcript has been reported to be reduced during hyphal induction in serum containing medium [22],[25],[26],[53]. We found that the decrease in *NRG1* expression during hyphal growth was dependent on the cAMP-PKA pathway, as Cyr1 (unpublished data) or Tpk2 was required for the reduction in *NRG1* expression (Figure 2C), consistent with the observation that *NRG1* transcript level was not down-regulated in the *efg1* mutant during hyphal induction in serum [26]. The transcriptional down-regulation was essential for the initiation of hyphal development, as ectopic expression of *NRG1* under the *MAL2* promoter in wild-type cells could not initiate hyphal development even under robust induction conditions (Figure 2D). However, the decrease in *NRG1* transcript alone is not sufficient to provide the temporal dynamic change in Nrg1 protein level during hyphal development. We observed that, by promoter shut-down experiments, Nrg1 was unstable when cells were inoculated into fresh medium at 30°C either from an overnight culture (Figure 2E) or a logarithmic growing culture (unpublished data). Adding farnesol inhibited Nrg1 degradation, suggesting that release from farnesol inhibition during inoculation is important in triggering the degradation of Nrg1. Therefore, the rapid and temporary disappearance of Nrg1 protein is the combined result of a cAMP-PKA dependent down-regulation of *NRG1* expression and a burst of Nrg1 degradation upon release from farnesol during inoculation. This temporary removal of Nrg1 is essential for hyphal induction.

### Promoter-Recruitment of Hda1 Is Required for Sustained Hyphal Development by Limiting Promoter Access to Nrg1

**Figure 3:**
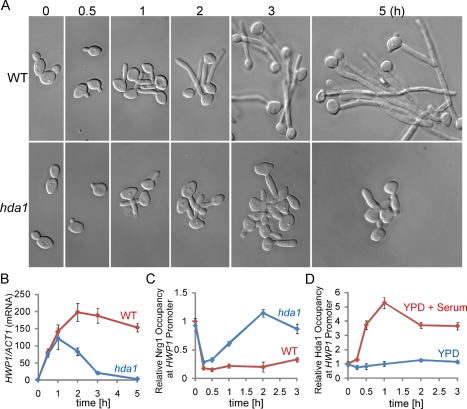
Promoter recruitment of Hda1 is required for hyphal maintenance by inhibiting Nrg1 access to the promoters of hypha-specific genes. (A, B) Hda1 is required for maintenance of hyphal development as shown by morphology and expression levels of hypha-specific genes during hyphal induction. Cells of wild-type (TS3.3+pBES116) and *hda1/hda1* (HLY4032+pBES116) strains were diluted into YPD with 10% serum at 37°C. *HWP1*, *ALS3*, *and ECE1* mRNA levels were determined by qRT-PCR. The signal obtained from *ACT1* mRNA was used as a loading control for normalization. (C) Nrg1-Myc binds the promoters of hypha-specific genes in *hda1* cells during hyphal elongation in YPD with 10% serum. (D) Serum-dependent binding of Hda1-Myc to the hyphal promoters in wild-type cells during hyphal induction. Cells of wild-type strain containing Hda1-Myc (HLY4033) were diluted into pre-warmed YPD medium at 37°C in the presence or absence of 10% serum. ChIP DNA in (C, D) were quantitated by qPCR with primers at the UAS regions of hypha-specific genes as described in Figure 1. Data for *ALS3* and *ECE1* from (B, C, D) are shown in Figure S2. All data show the average of three independent qRT-PCR or qPCR experiments with error bars representing the SEM.

One possible mechanism for the reduced promoter access by Nrg1 during hyphal elongation is a change in promoter chromatin. *C. albicans* has one class II HDAC Hda1 that functions as a repressor for phenotypic switching [54]. We found that cells deleted of *HDA1* were unable to maintain hyphal growth. They were impaired in sustained hyphal development and transcription of hypha-specific genes, but had no detectable defects during initial germ-tube formation (Figures 3AB, S2A and Table S1A). Furthermore, levels of promoter-bound Nrg1 increased dramatically in the *hda1* mutant after hyphal initiation, suggesting that Hda1 is required to prevent Nrg1 binding to the promoters for sustained hyphal growth (Figures 3C and S2B). These observations of the *hda1* mutant are similar to that of wild-type cells inoculated into rich medium at 37°C without serum (Figure 1). To determine whether Hda1 functions directly on the promoters of hypha-specific genes, we performed a time course ChIP of Hda1. We found that Hda1 was recruited to the promoters during hyphal induction in a serum-dependent manner (Figures 3D and S2C). We suggest that serum-induced Hda1 recruitment to the promoters of hypha-specific genes leads to a change in promoter chromatin that is no longer accessible to Nrg1, leading to sustained hyphal development.

In contrast to Hda1, we found that the Rpd3 HDAC preferentially associated with hypha-specific promoters in yeast cells and dissociated rapidly from the promoters upon hyphal induction, similar to the dynamic dissociation of Nrg1 (Figure S3). The sequential dissociation and recruitment of two different HDACs to the hypha-specific promoters likely account for the dynamic change in nucleosomal H4 acetylation levels during hyphal induction with the peak H4 acetylation at 30 min [50].

### The Promoter-Associated Hda1 Deacetylates the Yng2 Subunit of NuA4 Histone Acetyltransferase (HAT) module, Leading to the Reduction of Yng2 and NuA4 HAT at the Promoters of Hypha-Specific Genes

**Figure 4:**
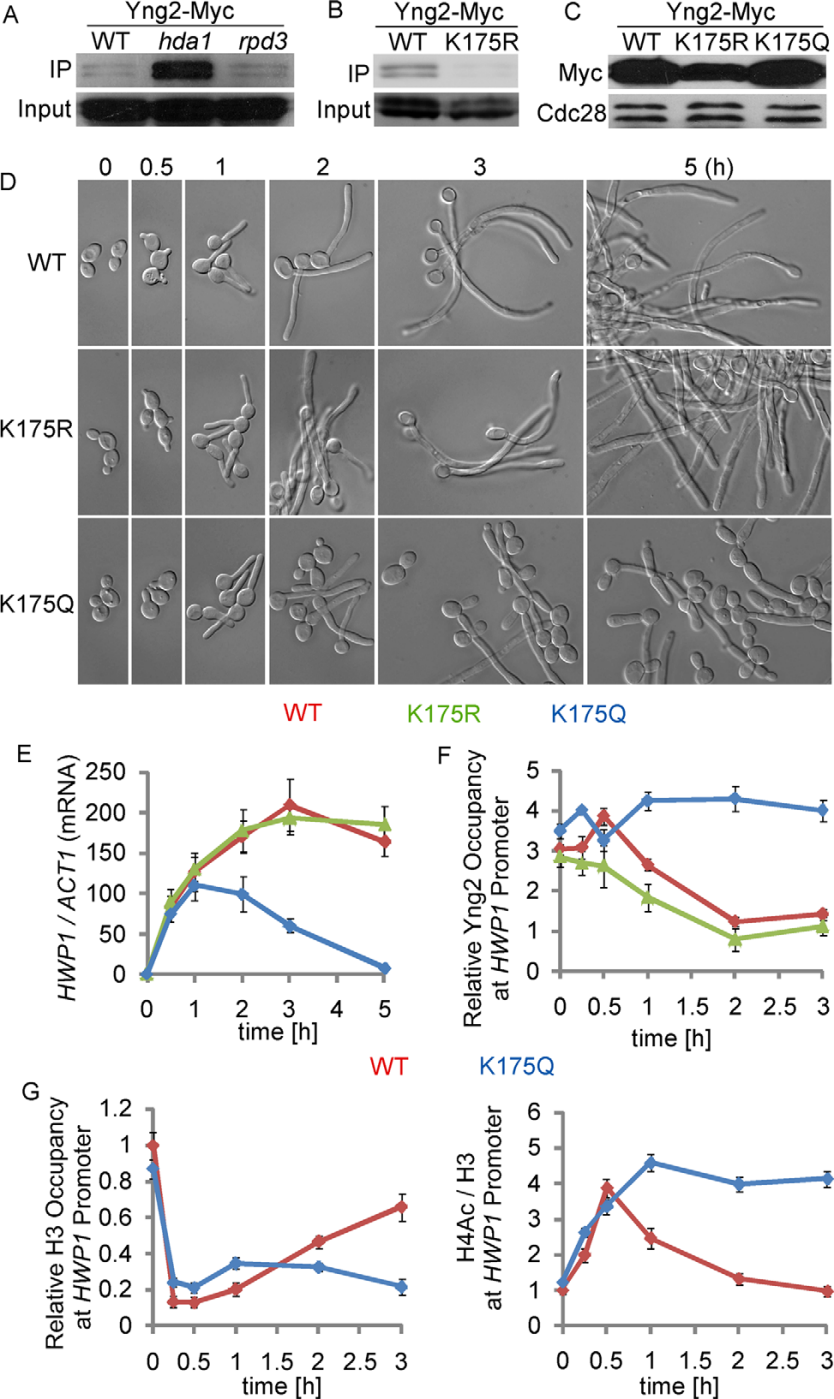
The function of Hda1 in sustained hyphal transcription is mediated through Yng2 deacetylation (A) Yng2p is deacetylated through an Hda1-dependent mechanism in vivo. Cells were grown at 30°C to OD_600_ 0.8, then WCEs were collected, immunoprecipitated with anti-Ac-K, and probed with anti-Myc. (B) K175 is the major acetylated lysine residue of Yng2 in vivo. Substitution of K175 with arginine (K175R) diminishes acetylation of Yng2. (C) Effects of K175 substitutions on Yng2 stability. K175R mutation causes decreased protein abundance of Yng2, while K175Q causes no detectable change. (D, E) Morphology and expression levels of hypha-specific genes in *YNG2* (HLY4035), *yng2^K175R^* (HLY4036), and *yng2^K175Q^* (HLY4037) cells during hyphal development in YPD with 10% serum at 37°C. *HWP1*, *ALS3*, and *ECE1* mRNA levels were determined by qRT-PCR. The signal obtained from *ACT1* mRNA was used as a loading control for normalization. (F) Dynamic dissociation of Myc-tagged Yng2 and yng2^K175R^ but not Yng2^K175Q^ from promoters during hyphal development by ChIP with anti-Myc antibodies. (G) ChIP with anti-H3 and anti-acetylated H4 antibodies show temporal dynamics in relative levels of H3 and H4 acetylation at the promoters of hypha-specific genes during hyphal induction in the *YNG2* (HLY4035) and *yng2^K175Q^* mutant (HLY4037) strains. Levels of H3 (relative H3 occupancy; bound/input) are normalized to the respective control DNA at the *ADE2* coding sequence region (bound/input). The 0-h values in the wild-type strain are set to be 1.00. Acetylation levels normalized with respect to H4 levels (H4 acetylation/H3 occupancy) are calculated by dividing the values for acetylated H4 with H3 occupancy values. Data on *ALS3* and *ECE1* for (E, F, G) are in Figure S4. All data show the average of three independent qPCR experiments with error bars representing the SEM.

How does promoter-associated Hda1 prevent Nrg1 from binding to the promoters of hypha-specific genes during hyphal elongation? One potential mechanism is chromatin remodeling. We have previously shown nucleosome reassembly and a decrease in H4 acetylation at the UAS regions of hypha-specific genes during hyphal elongation [50]. The decrease in H4 acetylation could be a result of H4 deacetylation by promoter-associated Hda1 or eviction of NuA4 from the promoters. *S. cerevisiae* Hda1 is a histone deacetylase specific for H3 and H2B [55]. Therefore, H4 may not be a substrate of the *C. albicans* Hda1. Yng2, a subunit of the NuA4 histone acetyltransferase HAT complex essential for HAT activity, is acetylated at lysine 170 by NuA4 and deacetylated by Rpd3 in *S. cerevisiae* [56]. Deacetylation of Yng2 leads to its degradation and eviction of chromatin-bound Yng2 with Esa1, the catalytic subunit of NuA4. In *C. albicans*, the NuA4 HAT complex is recruited to the promoters of hypha-specific genes [50]. By immunoprecipitation with anti-acetylated-lysine antibodies, we detected acetylated Yng2 in wild-type cells, but observed a dramatic increase in the level of acetylated Yng2 in the *hda1* mutant (Figure 4A). This suggests that deacetylation of Yng2 in vivo depends on Hda1 activity. K175 was identified as a candidate lysine residue for acetylation by sequence alignment with *S. cerevisiae* Yng2. This was confirmed by the loss of acetylation of Yng2 when we substituted K175 with arginine (K175R), a mutation that blocks acetylation (Figure 4B). The loss of acetylation of Yng2^K175R^ also suggests that K175 is the major acetylated lysine residue of Yng2 in *C. albicans*. The K175R mutation led to a reduced level of Yng2, whereas substituting K175 with glutamine (K175Q, a mutation mimicking constitutive acetylation) did not cause a detectable change of protein abundance relative to wild type (Figure 4C). Unlike the *yng2* deletion mutant, neither *yng2^K175R^* nor *yng2^K175Q^* has any detectable growth defect (unpublished data). We then examined whether Yng2 acetylation affects hyphal development. Both wild-type and *yng2^K175R^* cells developed long hyphae, whereas *yng2^K175Q^* cells were defective in sustained hyphal development and transcription (Figures 4D,E and S4A), a phenotype similar to that of the *hda1* mutant. Therefore, Yng2 deacetylation at K175 is important for sustained hyphal transcription. Conversely, constitutively acetylated Yng2 blocks hyphal filament extension.

**Figure 5:**
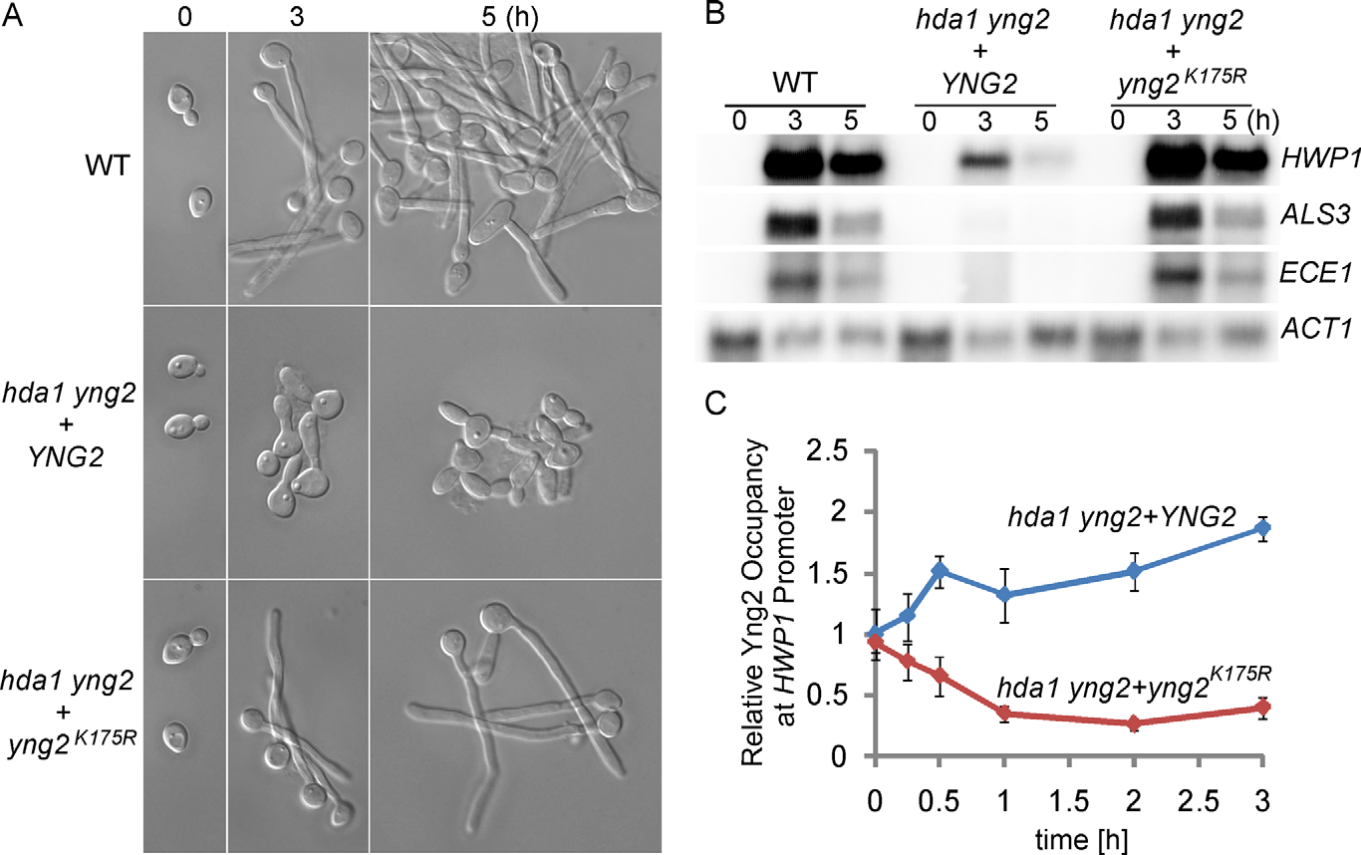
*yng2^K175R^* mutation suppresses the deficiency of *hda1/hda1* in sustained expression of hypha-specific genes. Morphology (A) and Northern analysis (B) of wild type and *hda1/hda1 yng2/yng2* transformed with *YNG2* or *yng2^K175R^* during hyphal induction as in Figure 3. Northern analysis was carried out using 12.5 µg RNA and probes to the indicated filament-specific transcripts. The *ACT1* transcript is included as a loading control. (C). Kinetics of Yng2-Myc and Yng2-Myc-K175R promoter binding in *hda1/hda1* mutants by ChIP with anti-Myc. Data on *ALS3* and *ECE1* for (C) are in Figure S5. The ChIP data show the average of three independent qPCR experiments with error bars representing the SEM.

If serum-induced Hda1 promoter association leads to Yng2 deacetylation and degradation, the level of promoter-associated Yng2 is expected to decrease during hyphal induction. Indeed, a time course ChIP of Yng2-Myc showed that Yng2 level decreased after 30 min of hyphal induction (Figures 4F and S4B). The level of promoter-associated Yng2^K175R^ showed a similar decrease, whereas the levels of Yng2-Myc in the *hda1* mutant and Yng2^K175Q^-Myc stayed unchanged (Figures 4F, 5C, and S4C). The level of Esa1, the enzymatic subunit of NuA4, showed a similar decrease at the promoters during hyphal induction (unpublished data).

To determine whether the deacetylation of Yng2 is the major function of Hda1 in hyphal development, we generated an *hda1 yng2* double mutant and introduced the *yng2^K175R^* mutation into the double mutant. The *hda1 yng2^K175R^* mutant behaved like *yng2^K175R^* or wild-type cells with no defect in hyphal filament extension and transcription (Figure 5A and 5B). Furthermore, Hda1 is required for the reduction of promoter-associated Yng2, and the *yng2^K175R^* mutant completely bypassed this requirement of Hda1 (Figures 5C and S5). Therefore, the function of Hda1 in sustained hyphal transcription is mediated completely through the deacetylation of Yng2 at K175. In addition, the level of promoter-associated Yng2^K175R^ decreased in the *hda1* mutant as did Yng2 in wild-type cells during hyphal elongation (Figures 5C and S5), suggesting that Hda1 deacetylation of Yng2 at the promoters led to the eviction of Yng2 and Esa1 from the promoters of hypha-specific genes.

To understand the effect of Yng2 K175 deacetylation on promoter chromatin, we determined temporal changes in histone levels and H4 acetylation at the UAS regions of hypha-specific promoters during hyphal induction (Figures 4G and S4D). Changes in H3 levels indicated rapid nucleosome disassembly during hyphal initiation and nucleosome reassembly during hyphal maintenance. The observed decrease in H3 occupancy and transcriptional induction within 30 min of hyphal induction is consistent with the notion that transcriptional activation correlates with reduced nucleosome occupancy [57]. In contrast, nucleosome reassembly during hyphal elongation seemed important for sustained hyphal transcription, as *yng2^K175Q^* cells showed a specific defect in nucleosome reassembly without any detectable impairment in initial nucleosome disassembly. Our data suggest that nucleosome reassembly during hyphal maintenance may function in preventing Nrg1 from binding to the promoters.

### Reduced TOR Signalling Led to Hda1 Promoter Recruitment and Sustained Hyphal Development

**Figure 6:**
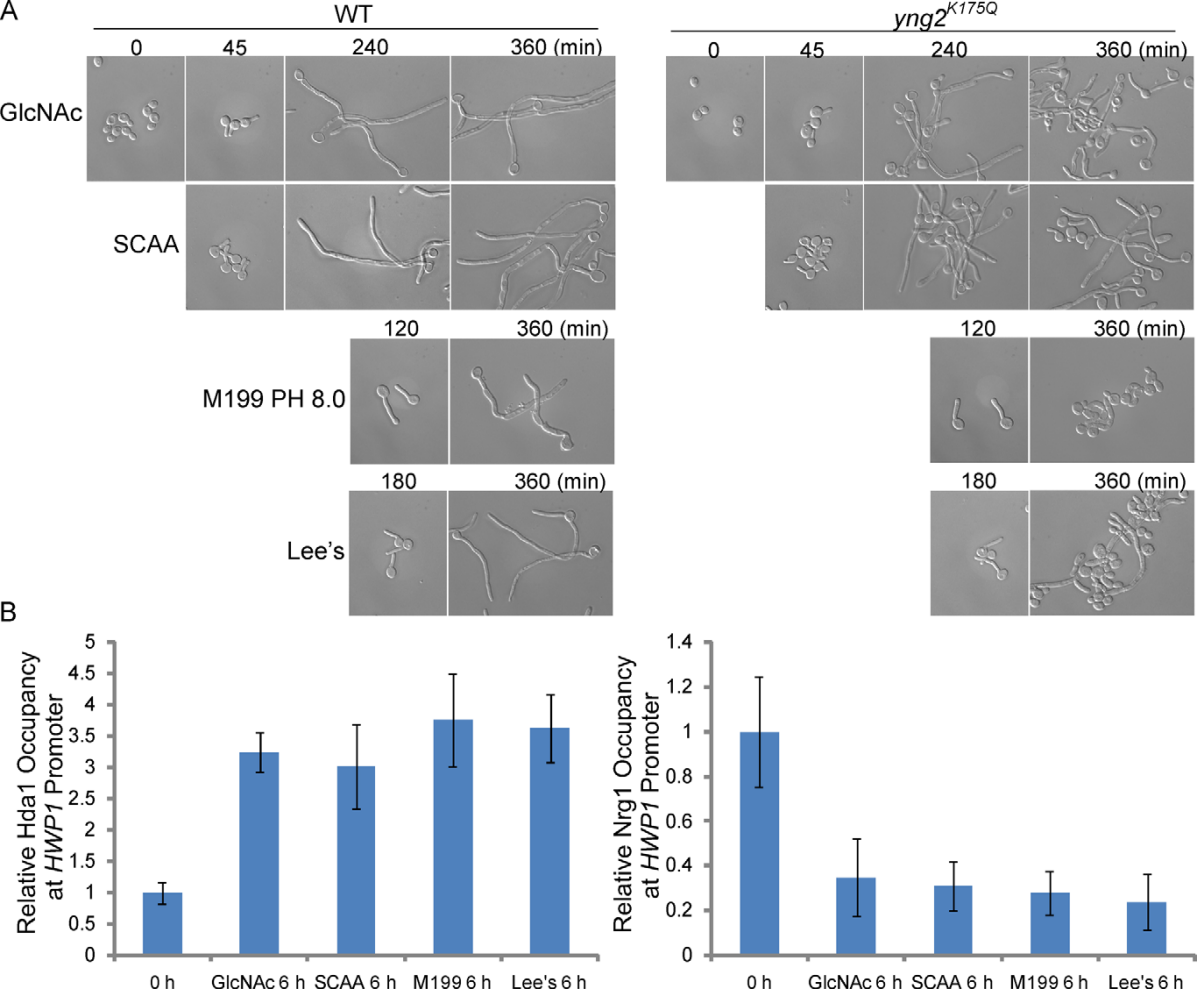
*yng2^K175Q^* mutant is defective in hyphal elongation in nutrient-poor media. (A) Cell morphology of wild-type (HLY4035) and *yng2^K175Q^* (HLY4037) cells grown at 37°C in YEP+GlcNAc, SCAA, M199 pH 8.0, or Lee's with mannitol for indicated times. (B) ChIP with anti-Myc antibodies in wild-type cells carrying Hda1-Myc (HLY4033) or Nrg1-Myc (HLY3922). qPCR was used to quantitate ChIP DNA with primers at the UAS region of *HWP1* under the indicated conditions. The ChIP data show the average of three independent qPCR experiments with error bars representing the SEM.

**Figure 7:**
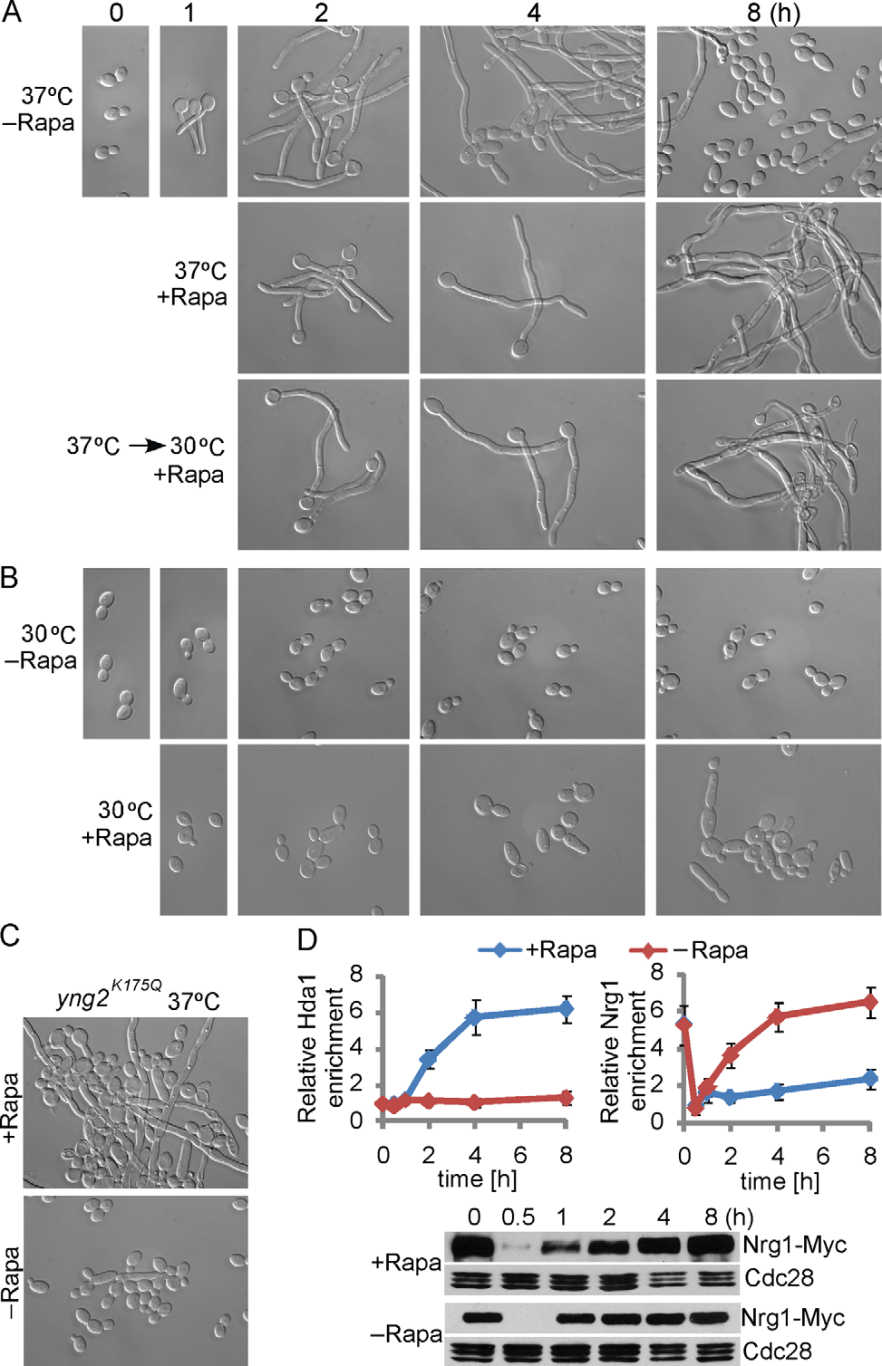
Reduced Tor1 signaling allows sustained hyphal elongation through promoter recruitment of Hda1. (A) Treatment of *C. albicans* cells with rapamycin after hyphal initiation at 37°C led to sustained hyphal filamentation in rich medium. Wild-type (HLY4035) cells were grown in YPD medium at 30°C overnight and were diluted at 1∶250 into pre-warmed YPD medium at 37°C or grown at 37°C for 1 h and then transferred to 30°C. 5 nM rapamycin was added after 1 h to half of the samples. Cells were collected at 0 h, 1 h, 2 h, 4 h, and 8 h for cell morphology. (B) Wild-type cells grown in YPD at 30°C with and without 5 nM rapamycin for the indicated hours. (C) *yng2^K175Q^* (HLY4037) cells were grown in YPD at 37°C for 8 h. 5 nM rapamycin was added after 1 h to half of the samples. (D) Nrg1 protein levels and Nrg1-myc or Hda1-myc promoter association during hyphal growth in YPD at 37°C with and without 10 nM rapamycin. ChIP DNA were quantitated by qPCR with primers at the UAS region of *HWP1*. The ChIP data show the average of three independent qPCR experiments with error bars representing the SEM.

*yng2^K175Q^* cells exhibited normal hyphal initiation in several hyphal growth media in addition to serum (Table S1B) and were defective in sustained hyphal development in all media that can support prolonged hyphal development (Figure 6A). This suggested that the Hda1-mediated deacetylation of Yng2 was required for hyphal maintenance in those media. In addition, Hda1 was found to be associated with the promoters of hypha-specific genes, whereas Nrg1 promoter-association was inhibited after 6 h of growth in those conditions (Figure 6B). Interestingly, those media tend to be nutrient-poor. One commonly used synthetic nutrient-poor medium for hyphal growth is Lee's medium [58]. It contains glucose, ammonium sulfate, and many amino acids, but lacks glutamine, a preferred nitrogen source. The rapamycin-sensitive Tor1 kinase is a central regulator of cell growth in response to nitrogen and amino acid availability in yeast [59] and is conserved in *C. albicans* [41]. We hypothesized that a nutrient-poor medium, such as Lee's, is sensed by *C. albicans* cells as nitrogen limiting, and therefore leads to reduced Tor1 activity. If so, treatment of *C. abicans* cells with rapamycin in a rich medium should mimic a nutrient-poor medium for sustained hyphal development. As predicted, treatment of *C. albicans* cells with a sublethal concentration of rapamycin after hyphal initiation at 37°C led to sustained hyphal filamentation in rich medium (Figure 7A). A higher concentration of rapamycin, however, slowed cell growth and hyphal elongation (unpublished data), consistent with the finding that rapamycin inhibits filamentation on nutrient-poor media [41]. Sustained hyphal filamentation was also observed at 30°C with sublethal levels of rapamycin after cells were first grown at 37°C for hyphal initiation (Figure 7A). The effect of rapamycin on hyphal maintenance was only observed after hyphal initiation. Cells treated with rapamycin at 30°C did not form hyphae (Figure 7B). We further showed that the effect of Tor1 on hyphal maintenance was mediated through the promoter-recruitment of Hda1, as *yng2^K175Q^* cells were defective in sustained hyphal filamentation in rapamycin (Figure 7C). In addition, Hda1 was recruited to the promoters of hypha-specific genes in cells treated with rapamycin in rich medium, whereas Nrg1 was excluded from the promoters (Figure 7D). The phenotypes of the *yng2^K175Q^* cells and patterns of promoter association for Hda1 and Nrg1 are similar between poor medium and rapamycin treatment (Figures 6 and 7). Furthermore, a mutant with a hyperactive TOR pathway in *C. albicans* has recently been shown to be unable to form hyphae in a nutrient-poor medium, and rapamycin can rescue the filamentation defect [60]. Therefore, maintenance of hyphal development requires reduced Tor1 signaling in response to nutrient-poor media and growth conditions. The reduced Tor1 signaling leads to the recruitment of Hda1 to the promoters of hypha-specific genes for promoter remodeling that prevents Nrg1 from binding.

### Temporal Connection between Hyphal Initiation and Maintenance

**Figure 8:**
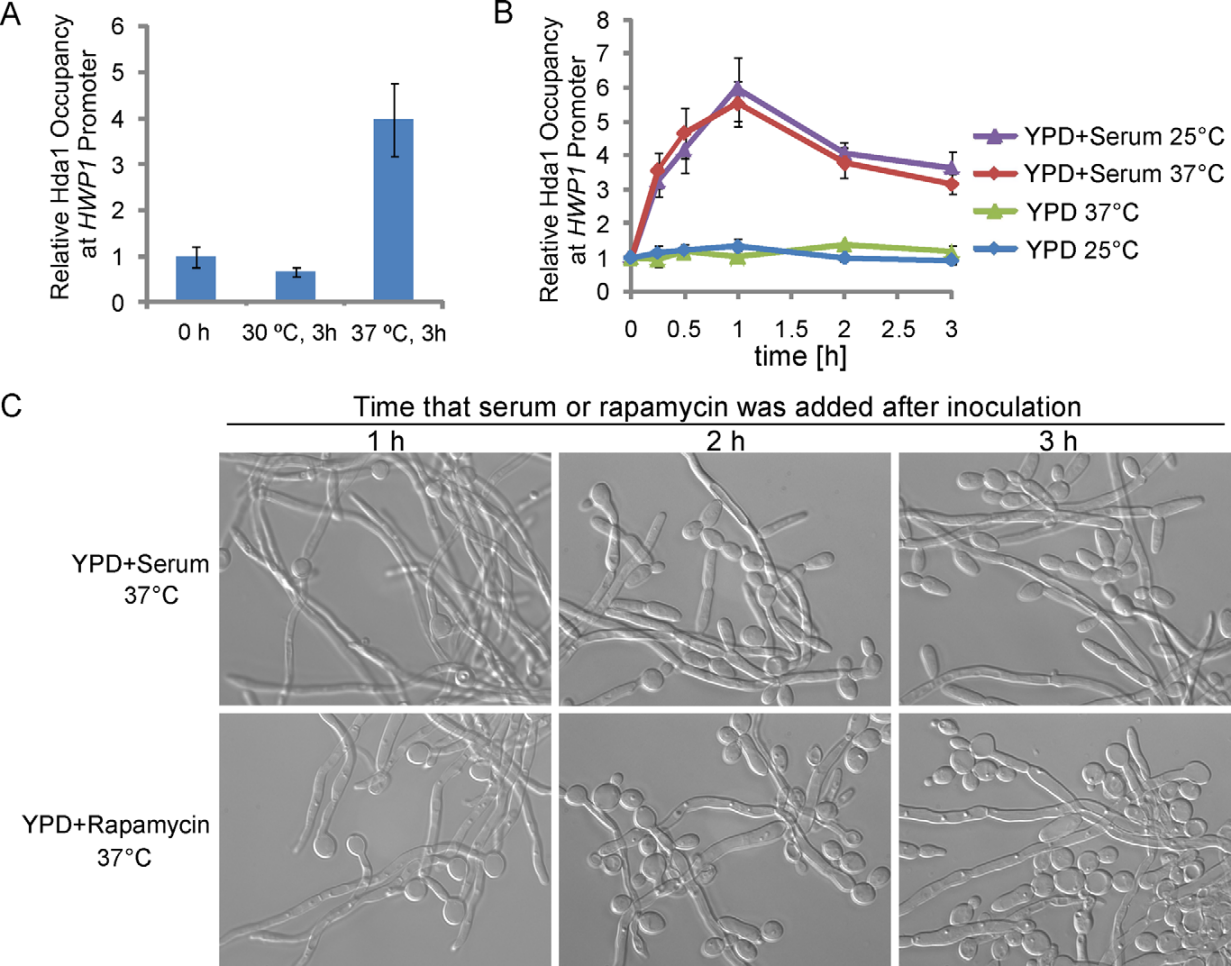
The sustained hyphal growth program could only be established during the time window of reduced Nrg1. (A) Serum is not sufficient to induce the promoter recruitment of Hda1 without hyphal initiation at 37°C. ChIP with anti-Myc antibodies in wild-type cells carrying Hda1-Myc in YPD with 10% serum under indicated conditions. Primers at the UAS region of *HWP1* were used. (B) Nrg1 removal is required and sufficient for serum-induced Hda1 promoter recruitment. The ChIP time course was carried out with anti-Myc antibodies in *nrg1/nrg1* cells carrying Hda1-Myc under the indicated conditions. Cells were collected at 0 min, 15 min, 30 min, 1 h, 2 h, and 3 h. The ChIP data show the average of three independent qPCR experiments with error bars representing the SEM. (C) Serum and rapamycin are unable to sustain hyphal development when they are added to YPD medium 2 h after inoculation at 37°C. Cells from overnight cultures in YPD at 30°C were diluted at 1∶250 into pre-warmed YPD medium at 37°C; 10% serum or 5 nM rapamycin were added at 1 h, 2 h, or 3 h after inoculation, respectively. The total incubation time is 5 h for serum and 8 h for rapamycin experiments.

To uncover potential molecular connections between the initiation phase and elongation phase of hyphal development, we asked whether serum could induce the promoter recruitment of Hda1 without the initiation phase. We found that Hda1 did not bind the promoters of hypha-specific genes when cells were grown at 30°C in the presence of serum (Figure 8A). Therefore, serum is not sufficient to induce the promoter recruitment of Hda1 without the initiation step. We further showed that raising the temperature to 37°C during inoculation was a prerequisite for subsequent recruitment of Hda1 to the promoters (Figure 8A). Since Nrg1 was temporally cleared when cells were inoculated into fresh medium at 37°C, a requirement for hyphal initiation, we then examined whether serum could induce promoter recruitment of Hda1 independent of temperature in the *nrg1* deletion mutant by a time course ChIP experiment. Hda1 was found to bind the *HWP1* promoter in the presence of serum in *nrg1* mutants in either 25°C or 37°C (Figure 8B). This result suggests that Nrg1 removal is required and sufficient for serum-induced Hda1 promoter recruitment for the sustained hyphal transcriptional program. This also predicts that the sustained hyphal transcriptional program could only be established within the time window of reduced Nrg1 when cells had just been inoculated into fresh media at 37°C. Indeed, addition of serum after 2 h of hyphal induction at 37°C in YPD could not sustain hyphal development (Figure 8C). Similar to serum, rapamycin could sustain hyphal growth only when added during hyphal initiation. Adding rapamycin after 2 h of hyphal induction showed no effect on hyphal maintenance (Figure 8C). Together, our data show a clear temporal connection between hyphal initiation and maintenance: Hda1 can be recruited to the promoters of the hypha-specific genes only when Nrg1 level is low. The time period of reduced Nrg1 during hyphal initiation can be viewed as a window of opportunity for establishing the hyphal transcription program. The strength and duration of the cAMP signal in each cell is expected to affect the commitment to the hyphal transcriptional program; once committed, the cAMP-PKA pathway activation is no longer needed. This is consistent with the observation that farnesol only inhibits germ-tube formation and has no effect on hyphal elongation [39]. The duration of hyphal development is determined by nutrient conditions through the TOR pathway.

## Discussion

**Figure 9:**
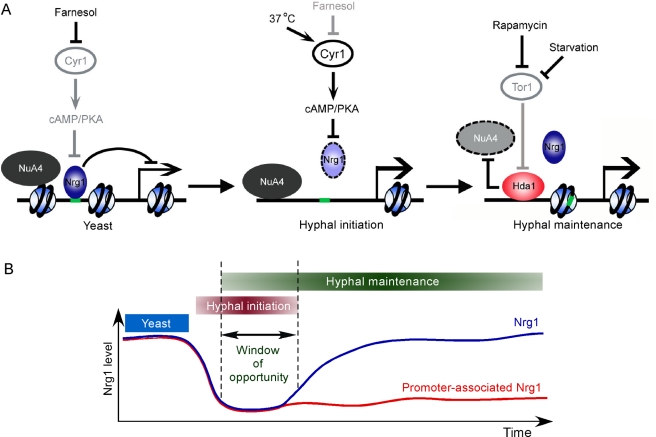
A schematic diagram depicting initiation and maintenance of hyphal development. (A) Hyphal development in *C. albicans* requires two phases of sequential changes in the promoter chromatin of hypha-specific genes. Initiation requires a rapid but temporary disappearance of Nrg1 in response to the activation of the cAMP-PKA pathway, while the duration of hyphal development is under the regulation of the TOR pathway by controlling promoter access to Nrg1 through recruitment of Hda1. Black lines represent active regulatory relationships; grey lines represent relationships that are inactive. Dashed circles represent degraded proteins. (B) Hda1 is recruited only during the time period when Nrg1 is absent. This provides a mechanism for temporal integration of two major nutrient-sensing cell growth pathways in development.

This study shows that the yeast to hyphae morphogenetic switch consists of two temporally related phases of regulation of the promoter chromatin of hypha-specific genes: the first for initiation and the second for maintenance of hyphal development (Figure 9). Initiation requires the cAMP-PKA pathway and maintenance requires reduced Tor1 signaling. A spike of cAMP-PKA activation [10] and release from the inhibition of quorum sensing molecules, such as farnesol, initiate hyphal development by transiently clearing Nrg1 protein. The duration of hyphal development is under the regulation of Tor1 by controlling promoter access to Nrg1 through promoter recruitment of Hda1. Therefore, hyphal maintenance requires active sensing of growth environments. Hyphal cells convert to yeast when nutrients are replete. This provides an underlying mechanism for the plasticity of dimorphism. Importantly, the sustained transcriptional program, as measured by promoter recruitment of Hda1, can only be established during the time period when Nrg1 is absent. This provides a temporal link at the molecular level between the two phases of hyphal development. This is the first example of a temporal integration of two major nutrient-sensing cell growth pathways in development. Temporal restriction in development of multicellular organisms is common. For example, nutritional control of the reproductive status in honeybees is restricted by a critical “decision-making” period in larval development [61]. Our study also provides a mechanism for how a transient signal, such as a cAMP spike, can make a long-lasting impact in transcriptional reprogramming during development. Such control of cellular development by a burst of proliferating signal, followed with sustained action of reduced TOR signaling has been observed for memory T-cell differentiation [62]. The TOR level is also critical for maintaining stem cell homeostasis [63]–[65]. Temporal regulation of cell fate by different signaling pathways is likely common in development of multicellular organisms. Coupling two signaling pathways through the regulation of promoter chromatin provides a unique mechanism.

Initiation of the hyphal transcriptional program requires temporary removal of Nrg1 protein. The timing, duration, and extent of the Nrg1 down-regulation are well correlated with timing and efficiency of hyphal initiation and are sensitive to multiple factors, including the state of inoculating cells (e.g., stationary cells), fold of dilution at inoculation, media, and temperature. Of those, a rise in temperature to 37°C and release from farnesol inhibition are essential for the clearance of Nrg1 and hyphal initiation. The cAMP-PKA pathway is required for the down-regulation of Nrg1, consistent with the concept that Cyr1 is a hub that integrates many of the hyphal inducing signals [31]–[33],[40]. Efg1 and Flo8 are both required for the down-regulation of Nrg1, but a T→A or T→E mutation at the sole conserved PKA site in Efg1 (T206) does not reduce or enhance hyphal gene expression (our unpublished data). If Efg1 activity is directly regulated by PKA phosphorylation, the regulation is not essential or sufficient for the induction of hypha-specific genes. Efg1 and Flo8 also function directly on the promoters of hypha-specific genes [16],[50], and the *tup1* mutant cannot bypass the requirement of Efg1 for hyphal transcription [66]. Our finding that Nrg1 is negatively regulated by the cAMP-PKA pathway and by farnesol is intriguing. The *nrg1* mutant produces 19-fold more farnesol than the parent wild-type strain [67], and farnesol in turn impacts activity of the Cyr1 pathway [40]. Together, these findings provide a loop of three negative feedback regulations from farnesol to Cyr1, to Nrg1, and then back to farnesol. Removal of farnesol during inoculation will lead to activation of the cAMP-PKA pathway and clearance of Nrg1 (Figure 9). Lack of Nrg1 in turn is expected to lead to increased production of farnesol, and a new balance among the components in the feedback loop will be achieved. Another intriguing finding is that the sustained hyphal transcriptional program can only be established during the time window of low Nrg1. Commitment to the hyphal program is therefore determined by the strength and duration of Nrg1 down-regulation in each cell. If the duration is not long enough to establish the Hda1-mediated chromatin remodeling, the cell will be in the yeast growth phase. This explains the cell-to-cell variation and experiment-to-experiment variation in hyphal induction in a given culture, and why yeast and hyphal cells can exist in the same culture. Our model explains a widely accepted observation that the quality of the initial hyphal induction is important for the fate of hyphal development. It also provides a molecular mechanism for why farnesol only inhibits germ-tube formation and has no effect on hyphal elongation [39].

Our finding of temporally coupled chromatin remodeling under reduced Tor1 signaling for sustained hyphal transcription provides a molecular mechanism for why nutrient-poor media are typical media for sustained hyphal development. It has been puzzling how *C. albicans* can respond to both rich and poor media and undergo hyphal development. Rich medium with serum is the most robust medium for hyphal induction, and this is consistent with the requirement of the cAMP-PKA pathway for hyphal growth. Hyphal induction in nutrient-poor media also requires an activation of the cAMP-PKA pathway, mostly through the release from farnesol inhibition. But sustained hyphal growth requires a nutrient limitation. Considering the conserved functions of Tor1 in nutrient sensing and growth regulation from yeast to human, involvement of Tor1 in hyphal development has been studied and implicated [41],[60],[68]. However, rapamycin cannot induce hyphal development, making it hard to reconcile if the cAMP-PKA and Tor1 pathways function in parallel. We find that rapamycin can lead to the promoter recruitment of Hda1 only during hyphal initiation in the absence of Nrg1. The temporal coupling of the two pathways through the regulation of the promoter chromatin of hypha-specific genes explains why rapamycin by itself cannot induce hyphae. Since serum can sustain hyphal development in YPD, we suspect it may contain certain components that are inhibitory to the Tor1 pathway. The nuclear localization and the activity of several nutrient-responsive transcription factors are regulated by Tor1-mediated phosphorylation in yeast [69]. It is possible that a Tor1-regulated transcription factor is necessary for the recruitment of Hda1 to the promoters of hypha-specific genes. Further studies are needed to address this. In addition to Hda1-mediated chromatin remodeling for sustained hyphal transcription, there is a built-in positive feedback on the hyphal transcriptional program that is also important for sustained hyphal development. The transcription factor Ume6, specifically expressed during hyphal development, controls the level and duration of hypha-specific genes and is important for hyphal elongation [53],[70],[71]. The strength of the cAMP and Tor1 signaling determines the level and duration of *UME6* expression, which in turn dictates the extent of hyphal morphogenesis. Hyphal morphogenesis and cell chain formation is under the control of another hypha-specific gene that encodes the G_1_ cyclin-related protein Hgc1 [72]–[76].

Our study should provide insights into understanding *C. albicans* pathogenesis. Hyphal initiation is tightly linked to release from quorum-sensing molecules, a condition parallel to dispersion of *C. albicans* cells from biofilms in human hosts, which is a major cause of disseminated candidiasis [77]. Invasive candidiasis is the most common invasive fungal infection among organ transplant recipients [78]. Rapamycin is given to transplant patients, especially renal transplant patients, to prevent organ rejection. One speculation from our finding is that rapamycin, known for its antifungal activity, may also facilitate hyphal development and invasive infections. The existence of extensive hyphae in various deep-seeded infection sites also suggests that those host environments may be perceived as stressful or starvation conditions by *Candida*. Our findings provide molecular mechanisms for how *C. albicans* adapts to varied host environments and develops from a benign commensal into a disseminated invasive disease.

We have previously noticed the dynamic increase and decrease in H4 acetylation concomitant with nucleosome disassembly and reassembly at the promoters of hypha-specific genes during hyphal induction [50]. The significance of the temporal dynamic regulation in promoter chromatin is not clear. Here we find the sequential dissociation of Rpd3 and association of Hda1 to the promoters is concomitant with the dynamic changes in H4 acetylation. Importantly, we show that promoter-associated Hda1 decreases H4 acetylation level by deacetylating Yng2 and evicting Yng2 and Esa1 of the NuA4 HAT module from the promoters, leading to nucleosome reassembly, inhibition of Nrg1 binding, and sustained hyphal transcription. This provides an example for Hda1 in gene activation and NuA4 in gene repression. The function of NuA4 in repression here is revealed by the use of *yng2^K175Q^* and *yng2^K175R^* mutations. Temporal analysis of promoter chromatin also delineates different functions of HDACs and NuA4 in transcription. It shows that nucleosome reassembly does not necessarily correlate with transcriptional repression; it can also be used for gene activation by restricting access of repressors. In *S. cerevisiae*, Yng2 is deacetylated by Rpd3 and a similar temporal dynamic H4 acetylation is observed at DNA double-stranded breaks concomitant with the sequential recruitment of NuA4 and Rpd3 [56]. Such temporal dynamic regulation of chromatin is likely also used in transcription in *S. cerevisiae*. Indeed Rpd3 is required for the expression of several environment-responsive genes [79]–[82]. Active functions of HDACs in gene expression are not limited to yeast. A recent genome-wide mapping of HAT and HDAC binding sites in human T cells shows that all HDACs analyzed are associated with active genes and positively correlated with transcription, with some HDACs mainly in the promoter regions and other HDACs in both the promoter and gene body regions [83]. Inhibition of HDAC activity with HDAC inhibitor treatment causes increases in acetylation in the active genes, suggesting that the majority of HDACs function to reset chromatin by removing acetylation at active genes [83]. The temporal dynamic NuA4 regulation by HDACs shown in this study provides an attractive mechanism likely used for temporal integration of different signals in transcriptional reprogramming during development.

## Materials and Methods

### Media and Growth Conditions

*C. albicans* strains were routinely grown at 30°C in YPD (2% Bacto peptone, 2% dextrose, 1% yeast extract). Transformants were selected on synthetic medium (2% dextrose, 0.17% Difco yeast nitrogen base w/o ammonium sulfate, 0.5% ammonium sulfate and auxotrophic supplements). M199 medium (Sigma-Aldrich) was buffered at pH 8 using 150 mM HEPES. Hyphal induction was performed as follows. Strains were grown overnight in liquid YPD at 30°C, pelleted, washed twice in PBS, resuspended in an equal volume of PBS, and diluted 1∶100, unless otherwise indicated, into YPD+10% serum (Sigma-Aldrich), Lee's medium [58] with modifications [5 g (NH_4_)_2_SO_4_, 2.5 g K_2_HPO4 Anhydrous, 5 g NaCl, 0.5 g alanine, 1.3 g leucine, 1 g lysine, 0.1 g methionine, 0.072 g ornithine, 0.5 g proline, 0.5 g threonine, 10 ml 20 mg/ml MgSO_4_, 1 ml 2 mg/ml biotin, with pH adjusted to 7.0, with either 1% glucose or mannitol, filled to 1 L with water], YEP+2% N-acetyl-glucosamine (Sigma-Aldrich), SCAA medium [15] with modifications [2% Casein Hydrolysate broth (Sigma-Aldrich), 0.17% Difco yeast nitrogen base w/o ammonium sulfate], Spider medium [1% mannitol, 1% nutrient broth, 0.2% K_2_HPO_4_, pH 7.2 before autoclaving] [84], M199 pH 8 or YPD+5 nM rapamycin. Cultures were grown at 37°C.

### Plasmid and Strain Construction

The *C. albicans* strains used in this study are listed in Dataset S1. Primer sequences are listed in Dataset S2. HDho15 and RPho19 [54] were streaked on 5-fluoro-orotic acid-containing medium to generate HLY4032 and HLY4039 (Ura^−^ strains for *hda1/hda1* and *rpd3/rpd3* mutants). Ura^+^ strains of TS3.3 [85] and HLY4032 were obtained by transforming *Asc*I digested pBES116 [37], which were used for hyphal induction. *hda1/hda1 yng2/yng2* mutants were generated by deleting *YNG2* in HLY4032 [50]. The disruption was confirmed by southern blotting (unpublished data).

A 0.9-kb PCR product (primers 1 and 2) containing the C-terminal *NRG1* coding region and a 1.3-kb PCR product (primers 9 and 10) containing the C-terminal *RPD3* coding region were inserted into the *BamH*I*-Mlu*I sites of pPR673 [50]. The resulting plasmids were digested with *Sac*I to target integration into their own loci to express Nrg1-13Myc and Rpd3-13Myc. A 1.3-kb PCR product (primers 5 and 6) containing the C-terminal *HDA1* coding region and a 0.8-kb PCR product (primers 7 and 8) containing the C-terminal *YNG2* coding region were digested with *Bgl*II and *Mlu*I and cloned into the *BamH*I-*Mlu*I sites of pPR673. The resulting plasmids were digested with *BamH*I to target the integration of the plasmid into its own locus to express Hda1-13Myc and Yng2-13Myc. The pMAL2-NRG1-13Myc plasmid was constructed by amplifying *NRG1-13MYC* (primers 3 and 4) from pACT1-NRG1-13MYC plasmid (our unpublished data). The PCR product was digested with *Xba*I and *Kpn*I to replace *WOR1-HA* from pMAL2-WOR1-HA plasmid [86]. The resulting plasmids were digested with *Asc*I to target integration into the *ADE2* locus to express Nrg1-13Myc.

The *YNG2* coding sequence was amplified using primers 13 and 14. The resulting 0.9-kb PCR product was digested with *Not*I and *Mlu*I and inserted into the *Not*I-*Mlu*I site of pPR673 to create pACT1-YNG2-13MYC. A 1.6-kb *YNG2* promoter fragment upstream of the START site of *YNG2* (from −1 to −1,600) was PCR amplified using primers 11 and 12. The resulting purified PCR product was digested with *Stu*I and *BamH*I and cloned into the *Stu*I-*BamH*I site of pACT1-YNG2-13MYC, displacing the 1-kb *ACT1* promoter region to generate pYNG2-YNG2-13MYC. Two-step PCR was used to create pYNG2-YNG2^K175R^-13MYC. Two pairs of primers (primers 13 and 15, 16 and 14) were used to PCR amplify overlapping *YNG2* fragments with the mutation in the overlapping region. The resulting PCR products were purified and mixed as templates for another round of PCR amplification using the primers 13 and 14, which produced the full-length *YNG2^K175R^* sequence. The resulting mutant, *YNG2^K175R^*, was cloned into the *Not*I-*Mlu*I site of the plasmid pYNG2-YNG2-13MYC, replacing the wild-type copy, and was confirmed by DNA sequencing. Two-step PCR was used to create pYNG2-YNG2^K175Q^-13MYC. Two pairs of primers (primers 13 and 17, and 18 and 14) were used to PCR amplify overlapping *YNG2* fragments with the mutation in the overlapping region. Subsequent steps were done by using the same methods as pYNG2-YNG2^K175R^-13MYC. These plasmids were digested with the *Pml*I within the *YNG2* promoter region for integration into the endogenous *YNG2* locus in *yng2/yng2* and *hda1/hda1 yng2/yng2* mutant strains.

### Northern and Quantitative RT-PCR Expression Analysis

Methods for RNA isolation and Northern blot hybridization were carried out as previously described [87]. Probes for *HWP1*, *ALS3*, *ECE1*, and *ACT1* were made by PCR amplification of ∼500-bp fragments from coding regions of each gene from SC5314 genomic DNA. The primers used were as follows: *HWP1*, primers 19 and 20; *ALS3*, primers 21 and 22; *ECE1*, primers 23 and 24; and *ACT1*, primers 25 and 26. For quantitative real-time reverse transcription-PCR (qRT -PCR) analysis, 10 µg of total RNA was DNase-treated at 37°C for 1 h using the DNA-free kit (Qiagen), cDNA was synthesized using the SuperScript II Reverse Transcriptase kit (Invitrogen), and qPCR was done using the iQ SYBR Green Supermix (Bio-Rad) using the primers 27 and 28 for *HWP1*, primers 29 and 30 for *ALS3*, primers 31 and 32 for *ECE1*, primers 33 and 34 for *ACT1*, and primers 35 and 36 for *NRG1*. The iCycler iQ detection system (Bio-Rad) was used with the following program: initial denaturation at 94°C for 5 min, followed by 40 cycles of 94°C for 20 s, 56°C for 30 s, and 68°C for 20 s. Amplification specificity was determined by melting curve analysis.

### Chromatin Immunoprecipitation

Chromatin immunoprecipitation was performed as described with modifications [50],[57]. Cells were formaldehyde cross-linked by adding formaldehyde (37%) to a 1% final concentration. Treated cultures were mixed by shaking and incubated for 15 min at room temperature. 2.5 M glycine was added to a final concentration of 125 mM, and treated cultures were mixed and incubated for 5 min at room temperature. Cells were pelleted at 3,000 *g* for 5 min at 4°C, washed four times with 20 ml PBS, and resuspended in 400 µl of 4°C lysis buffer (50 mM HEPES-KOH [pH 7.5],140 mM NaCl, 1 mM EDTA, 1% Triton X-100, 0.1% sodium deoxycholate) with protease inhibitors. All subsequent ChIP and wash steps were done at 4°C. Cells were lysed using a Fast-Prep system (FP120; Thermo Electron, Waltham, MA). DNA was sheared by sonication six times for 20 s at high power on a Bioruptor (diagenode) with 40 s intervals on ice.

For the IP, 10 µl of anti-Myc (SC-789, Santa Cruz), 4 µl of anti-H3 (ab1791; Abcam) or anti-acetylated-H4 (06-866; Millipore) antibodies were used for ∼4 mg of chromatin proteins in an immunoprecipitation volume of 200 µl. The IP was incubated O/N at 4°C, with agitation. Then 50 µl of a 50% suspension of protein A-Sepharose beads (GE Healthcare, 17-0974) in lysis buffer was added to the IP and incubated 2 h at 4°C with agitation. The beads were pelleted for 1 min at 3,000 g. After removal of the supernatant, the beads were washed with a series of buffers for 5 min each wash: twice in lysis buffer, twice in high-salt lysis buffer (50 mM HEPES-KOH [pH 7.5], 500 mM NaCl, 1 mM EDTA, 1% Triton X-100, 0.1% sodium deoxycholate), twice in wash buffer (10 mM Tris-HCl [pH 8.0], 250 mM LiCl, 0.5% NP-40, 0.5% sodium deoxycholate, 1 mM EDTA), and once in TE (10 mM Tris, 1 mM EDTA [pH 8.0]). After the last wash, 75 µl of elution buffer (50 mM Tris-HCl [pH 8.0], 10 mM EDTA, 1% SDS) was added to each sample, and the beads were incubated at 65°C for 10 min. The beads were spun for 1 min at 5000 g, and the supernatant was removed and retained. A second elution was carried out with 75 µl of elution buffer 2 (TE, 1% SDS) and eluates from the two elution steps were combined. For the ChIP input material set aside, 140 µl elution buffer 2 (TE, 1% SDS) was added to 10 µl WCE. ChIP and input samples were incubated overnight at 65°C to reverse the formaldehyde crosslinks. 150 µl of proteinase K solution (TE, 60 µg/ml glycogen, 500 µg/ml proteinase K) was added to each sample, and samples were incubated at 37°C for 2 h. Samples were extracted twice with 400 µl Tris buffer-saturated phenol/chloroform/isoamyl alcohol solution (25∶24∶1). 15 µl of 5 M NaCl and 0.8 ml of 100% ethanol (4°C) were added and the DNA was precipitated for 20 min at −20°C, then pelleted by centrifugation at 14,000 g for 10 min at 4°C, washed once with cold 70% ethanol, and allowed to air dry. The samples were resuspended in 30 µl of TE containing 100 µg/ml RNaseA and incubated for 1 h at 37°C. DNA derived from the whole cell extracts and immunoprecipitation (IP) eluate was analyzed by quantitative PCR (qPCR). Plotted are the average and standard error of the mean of three independent qPCR reactions for each experiment.

### Immunoprecipitation

Acetylated Yng2-Myc was immunoprecipitated with 30 µl protein A-sepharose beads (GE Healthcare, 17-0974) conjugated with 10 µl of rabbit polyclonal anti-acetylated-lysine (Cell Signaling, 9441S) at 4°C overnight in 200 µl pre-cleared WCEs, and detected with a peroxidase-conjugated anti-c-Myc antibody (Roche).

## Supporting Information

Figure S1 Nrg1 is detected in the nucleus of both apical and subapical cells of hyphae. Wild type cells carrying Nrg1-Myc (HLY3922) were processed for indirect immunofluorescence, as described [88]. Cells were grown in YPD+10% Serum medium at 37°C for hyphal induction or in YPD medium for yeast growth. Cells were fixed at 5 h after induction and stained for Nrg1-Myc with 9E10 mouse antibodies (Covance) and FITC-conjugated secondary antibodies (Jackson Laboratory). DNA was stained with DAPI. An untagged control (SC5314) was included.
(TIF)

Figure S2 Promoter recruitment of Hda1 is required for hyphal maintenance by inhibiting Nrg1 access to the promoters of hypha-specific genes. (A) *ALS3* and *ECE1* mRNA levels were determined by qRT-PCR as described in Figure 3 (B) Kinetics of Nrg1-Myc (B) and Hda1-Myc (C) binding at the *ALS3* and *ECE1* promoters were determined by ChIP as described in Figure 3C,D.
(TIF)

Figure S3 Rpd3-Myc disassociates rapidly from the promoters of hypha-specific genes upon hyphal induction, determined by ChIP as described in Figure 1B.
(TIF)

Figure S4 The function of Hda1 in sustained hyphal transcription is mediated through Yng2 deacetylation (A) *ALS3* and *ECE1* mRNA levels were determined by qRT-PCR as described in Figure 4(B) Relative Yng2 enrichment (B), relative H3 occupancy (C), and H4 acetylation level (D) at *ALS3* and *ECE1* promoter. ChIPs were performed as described in Figure 4E,F,G.
(TIF)

Figure S5 Kinetics of Yng2-Myc and Yng2^K175R^-Myc promoter binding in *hda1* mutants by ChIP with anti-Myc as described in Figure 5C.
(TIF)

Table S1 Yng2 deacetylation by Hda1 is not required for germ tube formation. (A) Germ tube formation of wild type and *hda1/hda1*. Cells of wild type (TS3.3+pBES116) and *hda1/hda1* (HLY4032+pBES116) were diluted 1∶100 into indicated medium at 37°C. The percentage of cells forming germ tubes in YPD+10% serum medium, Spider medium, and M199 pH 8 medium at 60 min or in Lee's medium at 180 min was determined by counting at least 300 cells/sample, in triplicate. The samples from Spider medium were gently sonicated to disrupt clumping. Mean (% germ tube formation) ± SE (standard error) of two independent experiments. The *hda1/hda1* mutant is able to form germ tube in YPD with serum and Spider media but shows a dramatically reduced level of germ tube formation in M199 and Lee's media. This is likely due to impaired growth of the mutant in the media. The doubling time of the wild type (TS3.3+pBES116) and *hda1/hda1* (HLY4032+pBES116) in YPD medium at 30°C is 105 min and 135 min, respectively, and in M199 PH 8 medium at 30°C is 150 min and over 18 h, respectively. The defect of *hda1/hda1* cells in germ tube formation in M199 is consistent with the report by Zacchi et al. [89]. (B) *yng2^K175Q^* mutant has no dramatic defect in germ tube formation. Cells of wild type *YNG2* (HLY4035), *yng2^K175R^* (HLY4036), and *yng2^K175Q^* (HLY4037) were diluted 1∶100 into indicated medium at 37°C. The percentage of cells forming germ tubes was calculated as described in (A). The two *yng2* mutants show a similar growth rate as the *YNG2* strain in all media examined.
(PDF)

## Abbreviations

ChIP: chromatin immunoprecipitation
HAT: histone acetyltransferase
HDAC: histone deacetylase
PKA: protein kinase A
TOR: target of rapamycin
