# Correction: Hyphal Development in *Candida albicans* Requires Two Temporally Linked Changes in Promoter Chromatin for Initiation and Maintenance

**DOI:** 10.1371/annotation/7b97b9ec-881a-4940-83ab-01f5318fd819

**Published:** 2011-12-13

**Authors:** Yang Lu, Chang Su, Allen Wang, Haoping Liu

Dataset S1 and Dataset S2 were erroneously omitted from the list of Supporting Information. Please view Dataset S1 here: http://www.plosbiology.org/corrections/pbio.1001105.s007.tif

and Dataset S2 here: http://www.plosbiology.org/corrections/pbio.1001105.s008.tif .

