# Supplementary figures and images for: Hyphal Development in Candida albicans Requires Two Temporally Linked Changes in Promoter Chromatin for Initiation and Maintenance
Source: PLoS Biol. 2011 Jul 19;9(7):e1001105. doi: 10.1371/journal.pbio.1001105 (PMC3139633; doi:10.1371/journal.pbio.1001105)

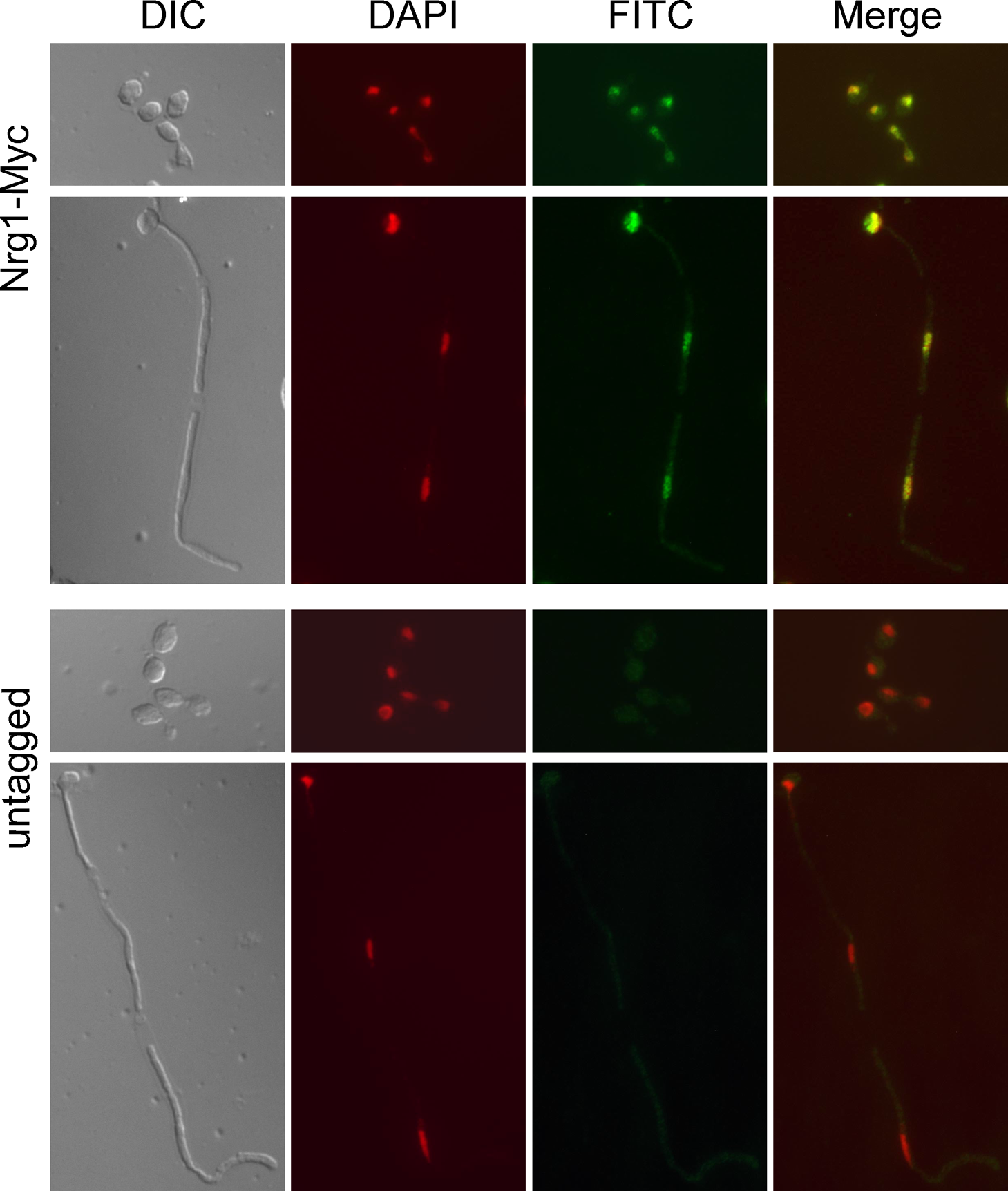

Supplement: Figure S1 — Nrg1 is detected in the nucleus of both apical and subapical cells of hyphae. Wild type cells carrying Nrg1-Myc (HLY3922) were processed for indirect immunofluorescence, as described [88]. Cells were grown in YPD+10% Serum medium at 37°C for hyphal induction or in YPD medium for yeast growth. Cells were fixed at 5 h after induction and stained for Nrg1-Myc with 9E10 mouse antibodies (Covance) and FITC-conjugated secondary antibodies (Jackson Laboratory). DNA was stained with DAPI. An untagged control (SC5314) was included. (TIF) [file pbio.1001105.s001.tif]

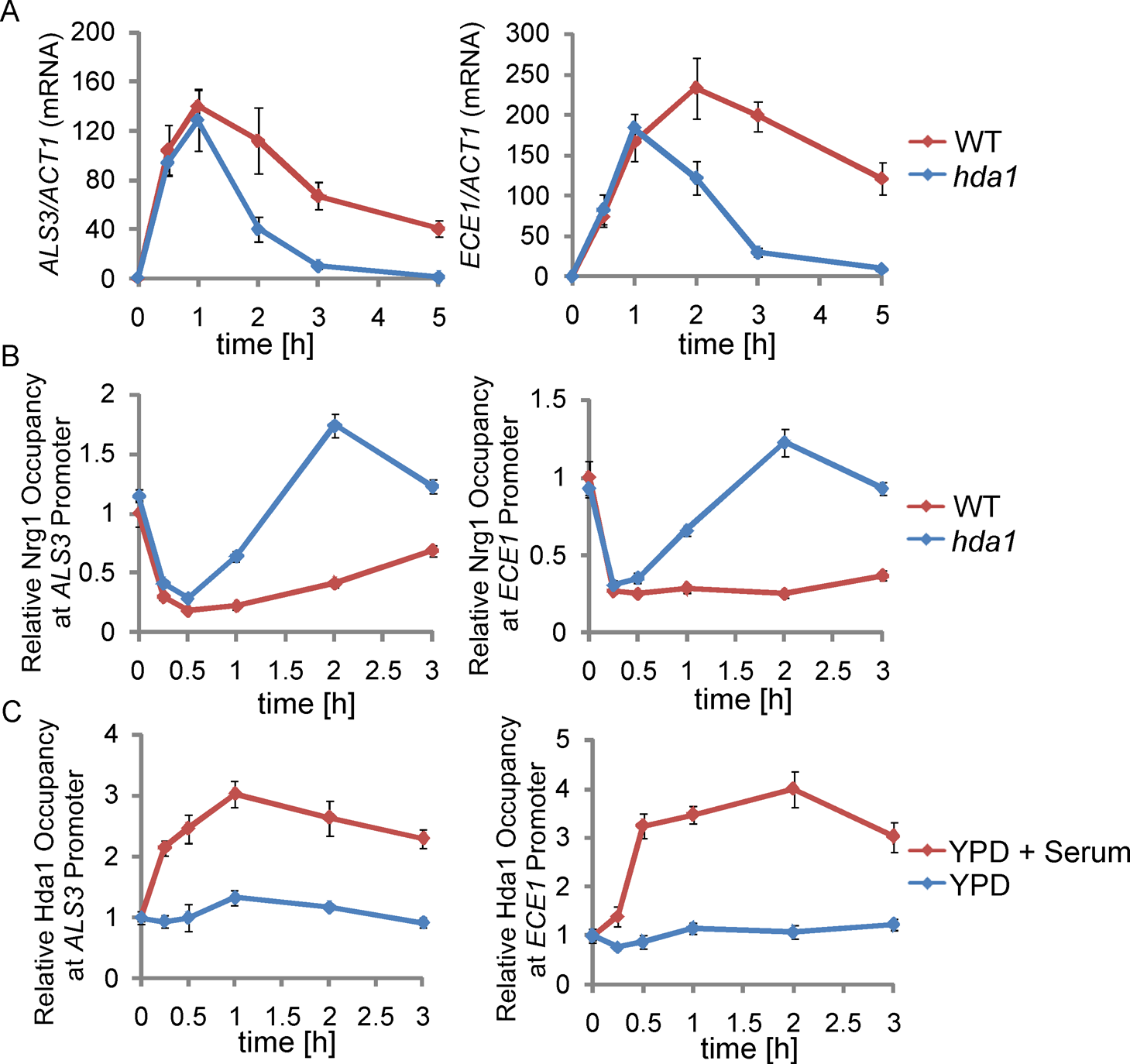

Supplement: Figure S2 — Promoter recruitment of Hda1 is required for hyphal maintenance by inhibiting Nrg1 access to the promoters of hypha-specific genes. (A) ALS3 and ECE1 mRNA levels were determined by qRT-PCR as described in Figure 3 (B) Kinetics of Nrg1-Myc (B) and Hda1-Myc (C) binding at the ALS3 and ECE1 promoters were determined by ChIP as described in Figure 3C,D. (TIF) [file pbio.1001105.s002.tif]

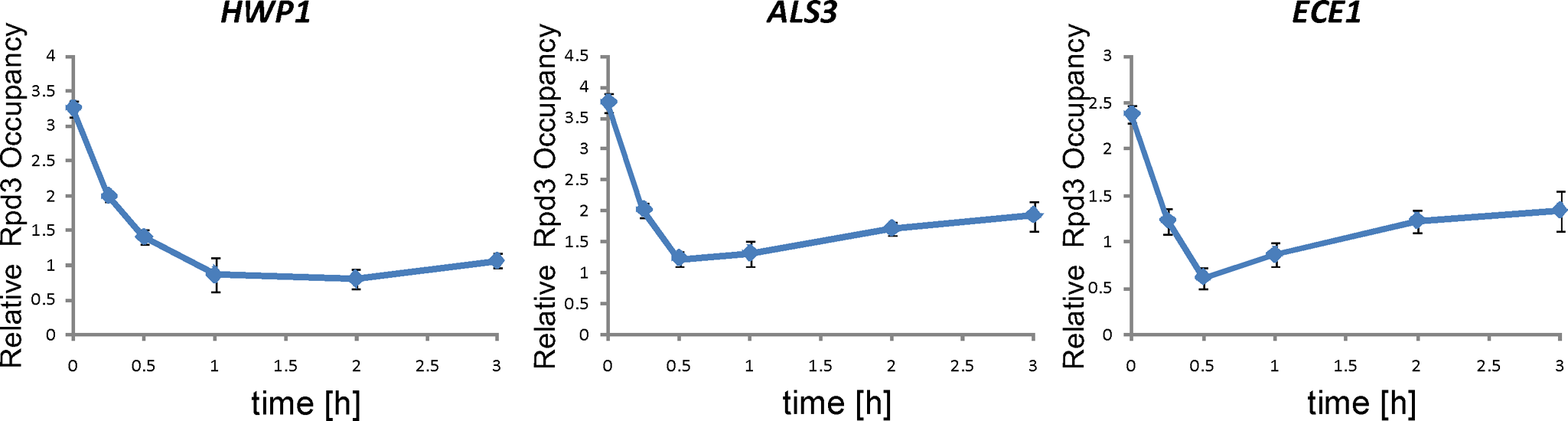

Supplement: Figure S3 — Rpd3-Myc disassociates rapidly from the promoters of hypha-specific genes upon hyphal induction, determined by ChIP as described in Figure 1B. (TIF) [file pbio.1001105.s003.tif]

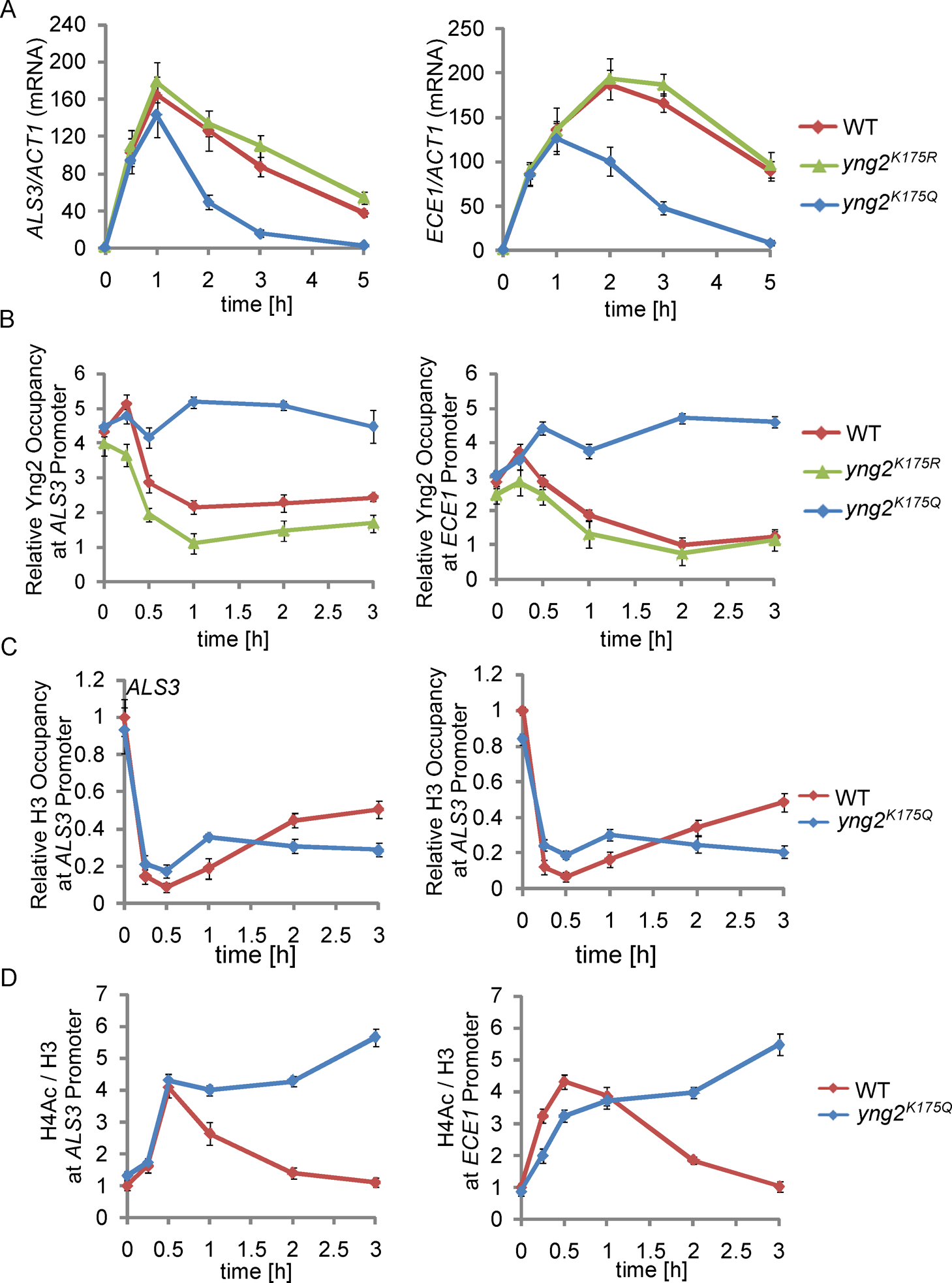

Supplement: Figure S4 — The function of Hda1 in sustained hyphal transcription is mediated through Yng2 deacetylation (A) ALS3 and ECE1 mRNA levels were determined by qRT-PCR as described in Figure 4(B) Relative Yng2 enrichment (B), relative H3 occupancy (C), and H4 acetylation level (D) at ALS3 and ECE1 promoter. ChIPs were performed as described in Figure 4E,F,G. (TIF) [file pbio.1001105.s004.tif]

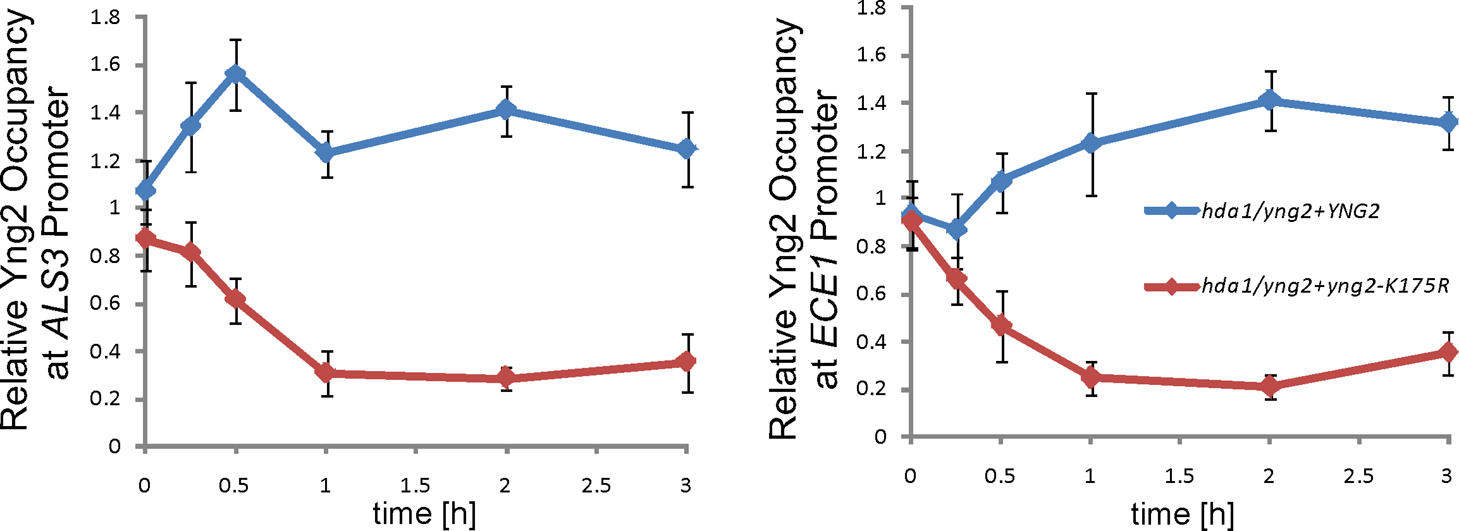

Supplement: Figure S5 — Kinetics of Yng2-Myc and Yng2K175R-Myc promoter binding in hda1 mutants by ChIP with anti-Myc as described in Figure 5C. (TIF) [file pbio.1001105.s005.tif]
