# Supplementary material for: Hyphal Development in Candida albicans Requires Two Temporally Linked Changes in Promoter Chromatin for Initiation and Maintenance
Source: PLoS Biol. 2011 Jul 19;9(7):e1001105. doi: 10.1371/journal.pbio.1001105 (PMC3139633; doi:10.1371/journal.pbio.1001105)
Supplement: Table S1 — Yng2 deacetylation by Hda1 is not required for germ tube formation. (A) Germ tube formation of wild type and hda1/hda1. Cells of wild type (TS3.3+pBES116) and hda1/hda1 (HLY4032+pBES116) were diluted 1∶100 into indicated medium at 37°C. The percentage of cells forming germ tubes in YPD+10% serum medium, Spider medium, and M199 pH 8 medium at 60 min or in Lee's medium at 180 min was determined by counting at least 300 cells/sample, in triplicate. The samples from Spider medium were gently sonicated to disrupt clumping. Mean (% germ tube formation) ± SE (standard error) of two independent experiments. The hda1/hda1 mutant is able to form germ tube in YPD with serum and Spider media but shows a dramatically reduced level of germ tube formation in M199 and Lee's media. This is likely due to impaired growth of the mutant in the media. The doubling time of the wild type (TS3.3+pBES116) and hda1/hda1 (HLY4032+pBES116) in YPD medium at 30°C is 105 min and 135 min, respectively, and in M199 PH 8 medium at 30°C is 150 min and over 18 h, respectively. The defect of hda1/hda1 cells in germ tube formation in M199 is consistent with the report by Zacchi et al. [89]. (B) yng2K175Q mutant has no dramatic defect in germ tube formation. Cells of wild type YNG2 (HLY4035), yng2K175R (HLY4036), and yng2K175Q (HLY4037) were diluted 1∶100 into indicated medium at 37°C. The percentage of cells forming germ tubes was calculated as described in (A). The two yng2 mutants show a similar growth rate as the YNG2 strain in all media examined. (PDF) [file pbio.1001105.s006.pdf]

A

	Mean (% germ tubes) ± SE (standard error)			
	YPD+ 10% Serum	Spider	M199 PH 8	Lee's Man
Wild type	92.7 ± 1.5	66.4±2.6	84.3±1.4	76.5±2.1
hda1/hda1	83.2±2.4	52.8±3.1	20.6±1.2	11.3±2.3

B

	Mean (% germ tubes) ± SE (standard error)			
	YPD + 10% Serum	Spider	M199 PH 8	Lee's Man
Wild type	96.8±1.9	76.7±2.4	87.4±3.2	83.3±1.8
$yng2^{K175R}$	97.4±1.4	79.2±2.8	90.5±1.6	84.8±2.1
$yng2^{K175Q}$	92.3±2.3	73.4±2.8	82.7±2.5	70.5±3.3
